# CDK9 and hematologic malignancies: pioneering novel therapeutic approaches

**DOI:** 10.1007/s10238-026-02141-3

**Published:** 2026-05-27

**Authors:** Hanie Raufi, Pouya Zahmatkesh, Sahar Safaei, Ata Bahadori, Saeed Solali

**Affiliations:** 1 Hematology and Transfusion Medicine Division, Department of Medical Biochemistry, Faculty of Medicine, Tabriz University of Medical Sciences, Tabriz, Iran; 2 Immunology Research Center, Tabriz University of Medical Sciences, Tabriz, Iran; 3 Molecular Medicine Research Center, Tabriz University of Medical Sciences, Tabriz, Iran

**Keywords:** Cyclin-dependent kinase 9 (CDK9), Hematologic malignancies, Transcriptional elongation

## Abstract

cyclin-dependent kinase 9 (CDK9) A key regulator of transcriptional elongation influences the transcription of oncogenes and anti-apoptotic proteins. Within the context of the positive transcription elongation factor b (P-TEFb) complex, CDK9 facilitates the phosphorylation of the C-terminal domain of RNA polymerase II. It also contributes to epigenetic regulation by phosphorylating histones and altering chromatin accessibility, thereby sustaining the transcription of oncogenic programs. This mechanism initiates the transcription of genes that are crucial for the proliferation and survival of cancer cells. A variety of hematological malignancies, including acute myeloid leukemia (AML), multiple myeloma (MM), and diffuse large B-cell lymphoma (DLBCL), have been associated with dysregulated CDK9 activity, which sustains oncogene expression and contributes to resistance to treatment. A notable therapeutic approach focuses on the inhibition of CDK9. This method may function as a therapeutic strategy that exploits the transcriptional addiction of malignant cells to short-lived oncogenic transcripts. By targeting a central regulator of transcriptional elongation, this approach selectively disrupts the sustained expression of survival-critical proteins such as MCL-1 and MYC, thereby exposing a therapeutic vulnerability in hematologic malignancies. Promising outcomes in preclinical and early clinical studies have been achieved by developing selective and pharmacokinetically more effective CDK9 inhibitors. Candidates for the combination of conventional chemotherapeutic drugs with other targeted therapy modalities. Research findings indicate that innovative CDK9 inhibitors and the silencing of CDK9 through siRNA may drastically change the therapeutic approach to hematological malignancies, particularly for patients facing recurrence or resistance to prior treatments. This review focuses primarily on hematologic malignancies because they exhibit pronounced transcriptional addiction to short-lived super-enhancer-driven transcripts (MYC, MCL-1), making CDK9 inhibition particularly effective. In contrast, solid tumors often display greater microenvironmental heterogeneity and compensatory pathways that limit single-agent efficacy.

## Introduction

 Despite therapeutic advances, numerous hematologic malignancies remain fundamentally driven by profound transcriptional dysregulation. A critical contributor to this phenomenon is the aberrant overexpression of CDK9, which fosters malignant cell survival by maintaining persistent transcription of pro-survival and proliferation-associated genes. This dysregulation synergizes with epigenetic alterations that remodel chromatin architecture, thereby sustaining the activation of oncogenic programs. CDK9 thus occupies a pivotal position at the intersection of epigenetic control and transcriptional dependency, enabling cancer cells to perpetuate unchecked growth and resist apoptotic cues [1]. While the primary focus of this review is on hematologic malignancies, CDK9 inhibition is also receiving growing attention in the field of solid tumors, such as breast, lung, and prostate cancers, where similar transcriptional dependencies have been identified, broadening its therapeutic potential across cancer types [2]. There is mounting evidence that the eukaryotic cell cycle is regulated by cyclin-dependent kinases (CDKs). CDKs phosphorylate their substrates to aid in DNA synthesis [3, 4]. CDKs phosphorylate a variety of substrates that coordinate the sequential events of the cell cycle, including DNA synthesis, chromosome segregation, and cytokinesis, beyond their classical role in cell cycle control, CDKs also participate in a range of vital biological functions which involve gene transcription, insulin signaling, and neural activity, highlighting their multifunctional nature [5]. A particular cyclin is attached to each CDK, directing the CDK’s activity. CDKs have been seen as attractive therapeutic targets because they regulate activities essential to the survival and proliferation of cancer cells. Indeed, numerous CDK inhibitors have been designed and synthesized and assessed across a range of malignancies [6]. A group of regulatory serine/threonine kinases known as the cyclin-dependent kinase (CDK) family comprises approximately twenty specific protein kinases that modulate critical cellular processes. Broadly, these kinases are categorized into two main functional subgroups: (i) CDKs involved in cell cycle regulation, such as CDK1, CDK2, CDK4, and CDK6, and (ii) CDKs primarily associated with transcriptional control, including CDK7, CDK8, CDK9, CDK12, and CDK13 [6]. Two primary transcriptional CDKs, CDK9/cyclin T and CDK7/cyclin H, phosphorylate specific serine residues on the carboxyl-terminal region of RNA pol II [7]. An essential step in transcription, RNA processing, and chromatin regulation is the phosphorylation of RNA polymerase II’s CTD [8]. For transformed cells to resist oncogene-induced apoptosis, RNA polymerase II must continue to function [9]. Recently, transcription-associated CDKs have gained a lot of attention from cancer researchers. operating through various layers of gene expression control, including transcription initiation, co-transcriptional splicing, and chromatin-based epigenetic modulation. this family of CDKs is essential for controlling gene expression. It is essential to develop particular pharmacologic inhibitors that target the cyclin-dependent kinase family [10]. Interestingly, these inhibitors were found to exert a more pronounced effect on malignant cells compared to their normal counterparts, non-transformed cells, even though they interfere with general transcriptional processes [11, 12]. CDK9 inhibition leads to a swift and coordinated suppression of numerous oncogenes, effectively impairing multiple critical oncogenic signaling pathways. Importantly, recent high-throughput profiling of regulatory networks has identified that many transcriptionally vulnerable genes are governed by a specialized class of super-enhancers. These regulatory elements are densely occupied by transcriptional machinery and are marked by high levels of active histone modifications, which contribute to their heightened transcriptional activity [13–15]. MCL1 can be indirectly targeted by CDK9 inhibitors. To take advantage of all messenger RNAs and proteins with short half-lives, CDK9 inhibition limits transcription elongation. Acute CDK9i therapy is effective against MCL1 and other targets with a short half-life, including MYC and other proto-oncogenes [16, 17]. Targeting CDK9 has emerged as a promising approach in cancer therapy. Over the past years, various pharmacological inhibitors of cyclin-dependent kinases (CDKs) have been developed, with several entering clinical trials. Among these, small-molecule CDK inhibitors (CDKIs) have exhibited the most promising agents for management of hematologic malignancies. This review aims to underscore the pivotal involvement of CDK9 in the development and progression of these malignancies by offering a comprehensive evaluation of its molecular structure, functional roles, and regulatory pathways. Additionally, it explores the current progress and therapeutic potential of pharmacological inhibitors targeting CDK9, highlighting their promise as emerging anticancer agents.

**Fig. 1:**
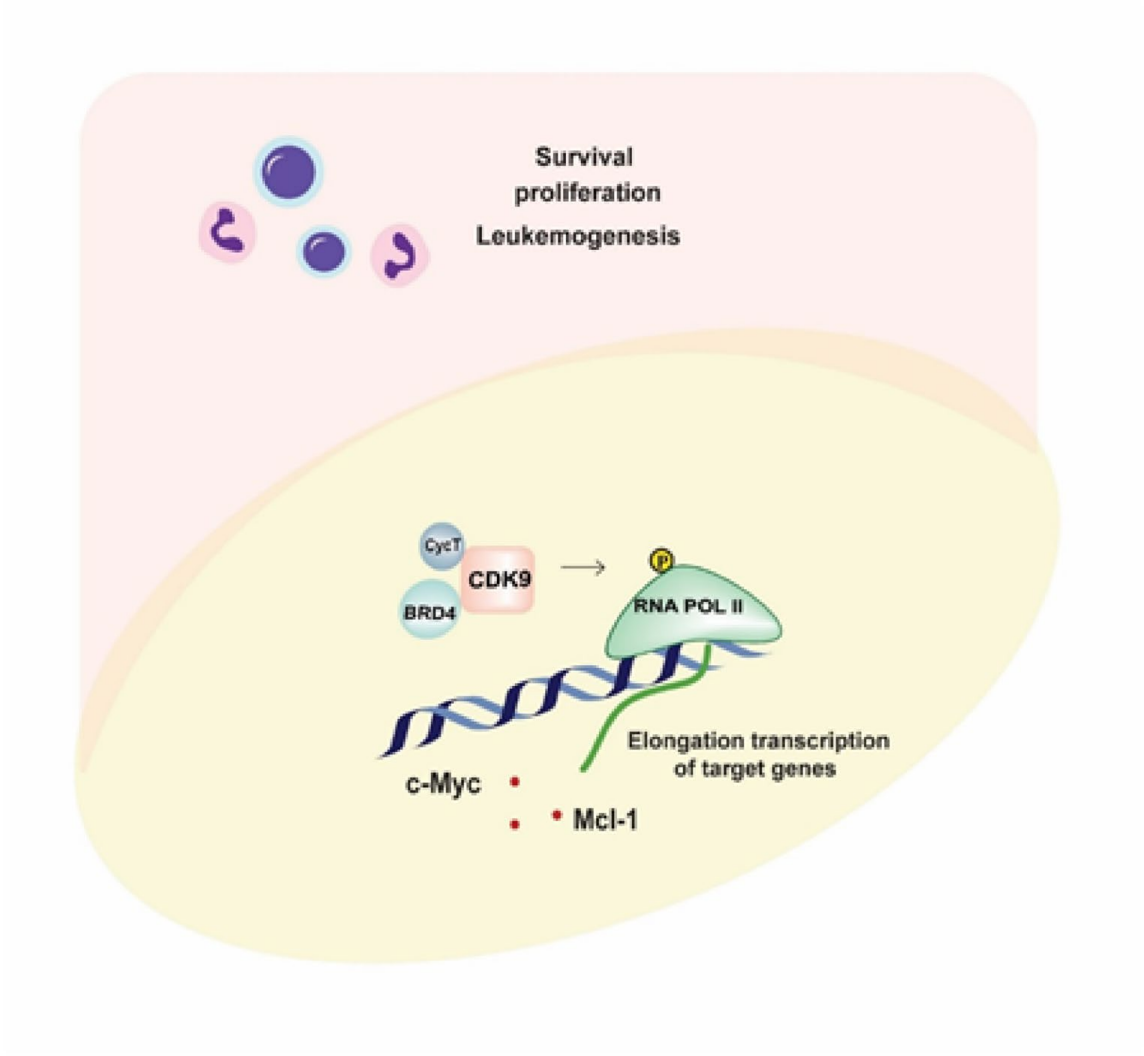
Central role of CDK9 in sustaining transcriptional addiction and leukemic cell survival. Schematic illustration of CDK9’s function in transcriptional elongation and leukemogenesis. As the catalytic component of the positive transcription elongation factor b (P-TEFb) complex, CDK9 associates with Cyclin T and is recruited to chromatin, in part through BRD4. Once activated, CDK9 phosphorylates the C-terminal domain (CTD) of RNA polymerase II at Ser2, releasing promoter-proximally paused polymerase and enabling productive transcriptional elongation. This process maintains the expression of short-lived oncogenic survival factors, including MYC and MCL1, thereby promoting leukemic cell survival, proliferation, and disease progression

## Cdk9

### Molecular structure of Cdk9

CDK9 shares the common structural features of cyclin-dependent kinases, comprising a bilobed architecture. The smaller N-terminal lobe (residues 16–108) contains a β-sheet and a single α-helix, while the larger C-terminal lobe (residues 109–330) is predominantly α-helical. The binding of Cyclin T1 is supported by a helix in the N-terminal region. A conserved ATP-binding site lies in the cleft between the two lobes, essential for kinase activity [18]. The ATP-binding site of CDK9, situated within the cleft between its two lobes, is essential for its catalytic function, as it facilitates the phosphorylation of substrate proteins. Moreover, the T-loop in the C-terminal lobe plays a regulatory role in CDK9 activation by undergoing phosphorylation at threonine 186, thereby promoting its involvement in transcriptional elongation. These key structural elements underscore CDK9’s potential as a promising target for the development of selective inhibitors in cancer treatment [19]. A nonpolar cavity situated at the interface of the N-terminal lobe’s β-sheet and the connecting loop specifically binds the adenine component of ATP [18, 20]. CDK9 is present in two isoforms: the 42 KDa isoform, which was initially identified and is more abundant, and the 55 KDa isoform, which is less abundant [21]. The promoter associated with the 42 kDa isoform displays significantly higher transcriptional activity than that of the 55 kDa isoform, making the shorter variant more abundantly expressed in most cell types. Functionally, both isoforms share the core kinase domain and participate in transcriptional regulation through phosphorylation of the RNA polymerase II C-terminal domain. However, the 55 kDa isoform contains an extended N-terminal region, which is thought to influence its cellular localization, stability, or interaction with regulatory proteins. These differences suggest that the two isoforms may have distinct roles in transcriptional control and cellular signaling, contributing to the complexity of CDK9-mediated gene regulation [22]. While CDK955 is mostly found to accumulate within the nucleolus, CDK9-42 is primarily found to be located within the nucleoplasm of the nucleus [21]. Further analysis revealed that the elevated expression levels of CDK9-42 observed in activated T cells, and monocytes can be attributed to the higher activity of its corresponding promoter [23, 24]. In certain physiological and pathological contexts (such as in resting macrophages, and cells responding to viral infections or extracellular stimuli) the 55 kDa isoform of CDK9 (CDK9-55) represents a larger fraction of total CDK9 protein. This suggests that the expression ratio of the two isoforms is dynamic and may vary depending on cell type, immune status, or environmental conditions [22].

### Biological function of CDK9

Eukaryotic RNA polymerase II (RNAPII) acts as the key enzyme driving the transcription of genes that code for proteins as well as many non-coding RNAs. The production of vital housekeeping genes is maintained under strict control, whereas genes involved in regulatory functions adjust their expression levels flexibly in response to various stimuli. This fine-tuned transcriptional control is essential for numerous cellular activities such as organismal development, managing stress, and promoting cell growth [25]. Protein synthesis through RNA polymerase II involves several steps, including initiation, promoter clearing, elongation, termination, mRNA cleavage, and polyadenylation, as well as co-transcriptional processing of nascent transcripts [26]. A group of general transcription factors (GTFs) called TFIIA, TFIIB, TFIID, TFIIE, TFIIF, and TFIIH work together to make the Pre-Initiation Complex (PIC). This is the first step in the process of RNA polymerase II starting transcription. RNA polymerase II is supported in this process by these GTFs [27, 28]. C-terminal domain (CTD) that plays a crucial role in Integrating the transcription process as well as the processing of mRNA as it is being synthesized. In both yeast and humans, this domain consists of multiple repeats of a seven-amino-acid sequence (26 repeats in yeast and 52 in humans) and is characterized by a flexible, unstructured configuration [29]. Transcription begins when the CTD is in a low-phosphorylation state. Shortly after initiation, the CDK7 kinase phosphorylates serine residues at positions 5 and 7 on the CTD, which helps to regulate the progression of transcription [30]. It is possible that CDK7 phosphorylates the RNA Pol II CTD at Ser5 as part of TFIIH-mediated promoter release [31]. At the beginning of transcription, RNA polymerase II produces a precursor transcript around fifty ribonucleotides long from the promoter. However, it stops producing the transcript because of two negative regulators working together: first, NELF; second, DSIF [32]. The CDK9-cyclin T complex, commonly Identified as positive transcription elongation factor b (P-TEFb), required for robust gene expression. It exerts its effect by phosphorylating the C-terminal domain (CTD) of RNA polymerase II, as well as transcriptional pausing factors such as DSIF and NELF. This phosphorylation relieves promoter-proximal pausing and facilitates the transition of RNA polymerase II into productive elongation, thereby enhancing transcription efficiency. Capping of 5′ messenger RNA by the human capping enzyme, RNA quality control, and gene-specific regulation are all ensured by transcriptional pausing. Once RNA Pol II is halted, the PTEFb/CDK9 complex is brought to the complex, where it works in tandem with BRD4, together with the super elongation complex (SEC), plays a crucial role in releasing RNA polymerase II from pausing, enabling it to engage in active transcription elongation. This process not only supports efficient transcriptional elongation but also facilitates the recruitment of essential factors involved in 3′-end processing and splicing, which are critical for producing mature messenger RNA (mRNA) ready for translation. CDK9 contributes to these events by phosphorylating the C-terminal domain (CTD) of RNA polymerase II, as well as the transcriptional pausing factors DSIF and NELF, thus enabling the shift from a paused transcription state into active and efficient RNA synthesis [33, 34]. Many activators that bind to DNA use P-TEFb to mediate the effect of their enhancers. The following are included: NF-kB, c-Myc, STAT 3, MyoD, MEF2, and hormone receptors (estrogen and androgen receptors) are included [35]. In the beginning, researchers focused on studying certain transcription components, such as c-myc, HIV, and Hsp genes, to understand how to affect transcription elongation [36]. However, further genome-wide studies have shown that stalled RNA polymerase II is already positioned at the promoters of many dormant and inducible genes [37, 38]. As a result, Positive transcription elongation factor b (P-TEFb) plays a central role in regulating the transcription elongation phase emerges as a key control point in eukaryotic gene expression. While P-TEFb plays a critical role in promoting early transcription elongation, other CDKs that phosphorylate serine 2 (S2) residues within the C-terminal domain (CTD) of RNA polymerase II also contribute to later stages of transcription. For instance, CDK12, in complex with cyclin K (CycK), facilitates transcriptional termination and mRNA processing, including cleavage and polyadenylation (CPA), by recruiting CPA factors through interactions with SR proteins and the PAF1 complex (PAF1C), which recognize the phosphorylated CTD [39, 40]. Nascent RNAs, and stability of recently synthesized RNA molecules largely rely on the activity of CDK9 and the control systems linked to it. Continuous transcription of super-enhancers may be essential for **c**ell endurance and replication. Consequently, targeting the transcriptional machinery in cancer cells could selectively suppress genes critical for maintaining their malignant state [41]. Molecularly targeted therapies are designed to minimize non-specific cytotoxic effects on normal cells by selectively targeting proteins that are overexpressed or dysregulated in cancer cells. Consequently, many small-molecule inhibitors developed to date have focused on oncoproteins as their primary targets. However, a growing number of recently developed agents exhibit potent anti-tumor activity by acting on components of the general cellular machinery, rather than on cancer-specific proteins alone. Among the newly developed drugs are small**-**molecule inhibitors targeting, CDK9, and BRD4, that inhibit gene expression by disrupting the activity of RNA polymerase II [11, 12]. According to data from cBioPortal, CDK9 is overexpressed across a wide range of malignancies, highlighting its potential significance in tumor biology. This elevated expression suggests that CDK9 may play a critical role in supporting the uncontrolled growth and survival of cancer cells. In addition to its widespread dysregulation in cancer, CDK9 possesses several properties that make it a compelling target for therapeutic intervention — including its central role in transcriptional regulation, particularly in controlling the elongation phase via RNA polymerase II, and its involvement in the expression of oncogenes and anti-apoptotic genes. These features collectively position CDK9 as a promising candidate for the development of novel cancer therapies aimed at selectively disrupting transcriptional programs that are essential for malignancy [42]. CDK9 activity is tightly controlled through a dynamic equilibrium between its active form within the positive transcription elongation factor b (P-TEFb) complex and its inactive sequestration within the 7SK small nuclear ribonucleoprotein (7SK snRNP) complex [43]. In resting conditions, a substantial fraction of P-TEFb is bound to 7SK RNA together with HEXIM proteins, rendering it catalytically inactive [44]. Oncogenic signals, including those mediated by BRD4, MYC, or super-enhancer–associated complexes, promote the release of CDK9/Cyclin T1 from 7SK snRNP, shifting the balance toward active P-TEFb [45]. This redistribution enhances phosphorylation of RNA polymerase II CTD at Ser2, facilitating pause release and productive elongation [46]. In hematologic malignancies, constitutive recruitment of P-TEFb by aberrant transcriptional programs establishes sustained oncogene transcription and reinforces transcriptional dependency [47]. Beyond its canonical role in RNA polymerase II pause release, emerging evidence indicates that CDK9 also modulates chromatin architecture and epigenetic states, thereby extending its regulatory influence from transcriptional elongation to broader chromatin-based control mechanisms.

**Fig. 2:**
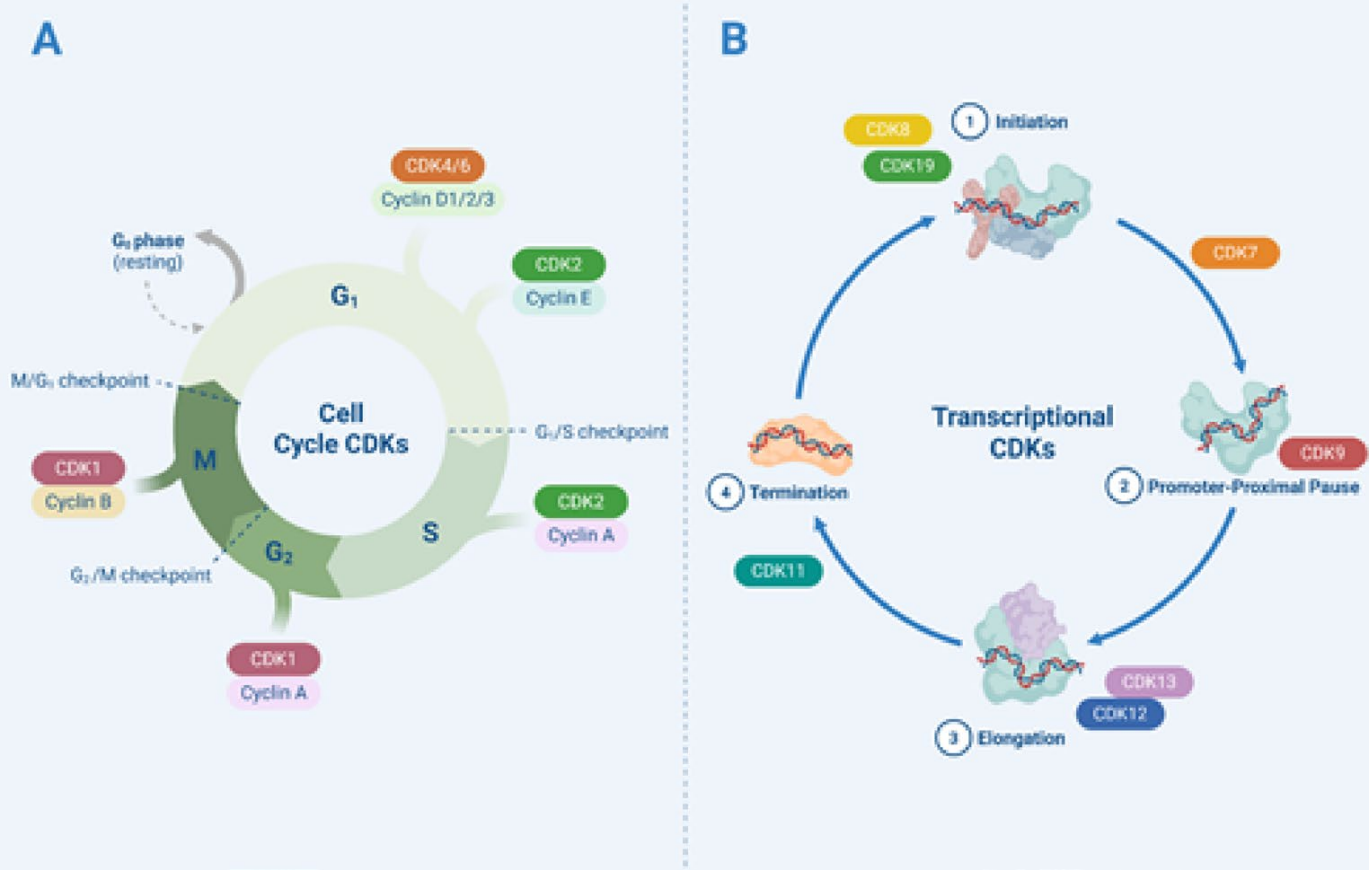
Functional classification of cyclin-dependent kinases (CDKs) involved in cell cycle regulation and RNA polymerase II–dependent transcription. (**A**) Schematic overview of classical cell cycle–associated CDKs and their cognate cyclin partners that regulate progression through distinct phases of the cell cycle. CDK4/6–cyclin D complexes control G1 phase progression, CDK2–cyclin E and CDK2–cyclin A regulate the G1/S transition and S phase, and CDK1, in association with cyclin A or cyclin B, governs the G2/M transition and mitosis. Major cell cycle checkpoints are indicated., (**B**) Overview of transcription-associated CDKs that coordinate successive stages of RNA polymerase II–mediated transcription. CDK7, as a component of the TFIIH complex, regulates transcription initiation and C-terminal domain, (CTD) priming. CDK8 and CDK19, functioning within the Mediator kinase module, modulate transcriptional output and promoter-proximal pausing. CDK9, the catalytic core of the P-TEFb complex, phosphorylates the RNA polymerase II CTD at Ser2 as well as negative elongation factors, thereby releasing paused polymerase into productive elongation. CDK12 and CDK13 further support transcriptional elongation and participate in RNA processing, while CDK11 contributes to late transcriptional events, including RNA processing and termination. Collectively, these CDKs orchestrate the precise temporal control of gene expression

### Roles of CDK9 in epigenetic silencing

The structural configuration of chromatin is fundamentally important in regulating gene activity within eukaryotic cells. Chromatin generally exists in two distinct organizational states: euchromatin and heterochromatin. Euchromatin exhibits a loosely packed configuration, which permits transcription factors and RNA polymerase to access DNA and initiate gene transcription. On the other hand, heterochromatin remains in a densely compacted state, limiting DNA accessibility and thereby suppressing transcriptional activity [48]. The epigenetic modifications that take place in cancers are the ones that are responsible for the suppression of tumor suppressor genes, also known as TSGs. More specifically, methylation in TSG promoter segments facilitates the recruitment of repressor complexes, which in turn leads to the development of heterochromatin [49]. By phosphorylating BRG1, a core subunit of the chromatin remodeling complex, CDK9 plays a critical role in preserving transcriptional repression in cancer cells. This complex is responsible for recruiting BRG1 to heterochromatin, where it moves and restructures nucleosomes and mediates gene transcription. Importantly, experimental evidence indicates that targeting CDK9 causes BRG1 to become dephosphorylated, enabling it to interact with chromatin and remodel it, which ultimately leads to the reactivation of TSGs [49]. Similar to how DNMT inhibitors (DNMTi) demethylate the promoters of silenced TSGs to reactivate their expression [50], Zhang et al. demonstrated that inhibiting CDK9 produces a comparable effect on transcription. Therefore, combining CDK9 inhibition with DNMT inhibition could synergistically promote the reactivation of TSGs and help prevent tumor progression. These findings expand the therapeutic implications of CDK9 inhibition beyond suppression of short-lived oncogenes. By facilitating BRG1 recruitment and tumor suppressor gene reactivation, CDK9 inhibitors may synergize with epigenetic therapies such as DNA methyltransferase inhibitors or BET inhibitors. This dual transcriptional and chromatin-remodeling effect reinforces the rationale for rational combination strategies in hematologic malignancies.

**Fig. 3:**
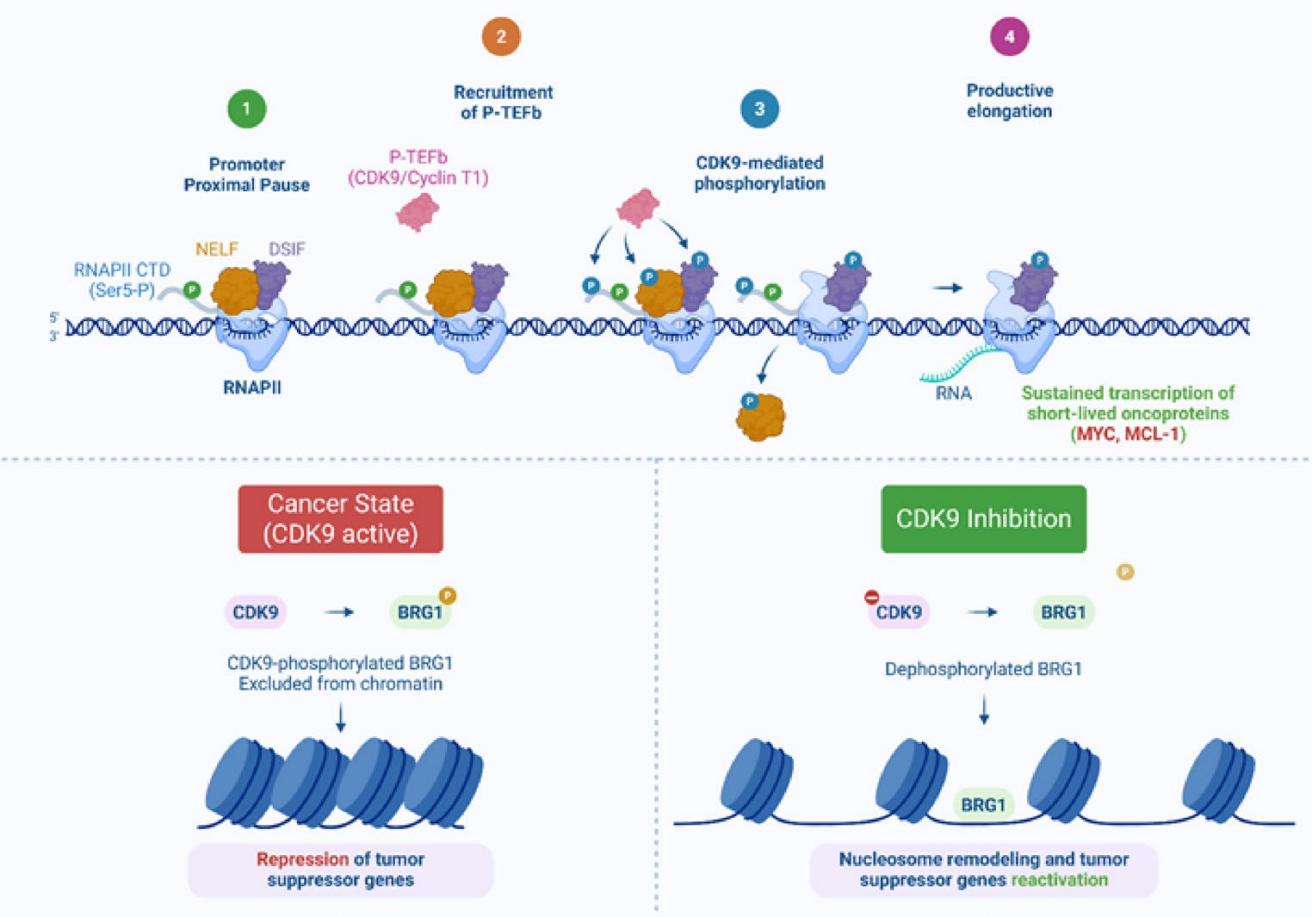
Core transcriptional and epigenetic functions of CDK9. CDK9, as a component of the P-TEFb complex, phosphorylates the RNAPII C-terminal domain at Ser2, as well as DSIF and NELF, thereby releasing promoter-proximal pausing and promoting productive transcriptional elongation. This activity sustains the expression of short-lived oncogenic proteins, including MYC and MCL-1. Beyond its role in transcriptional regulation, CDK9 also influences chromatin dynamics through phosphorylation of BRG1. In cancer cells, BRG1 phosphorylation leads to its exclusion from chromatin, thereby maintaining repression of tumor suppressor genes. In contrast, CDK9 inhibition facilitates BRG1 recruitment to chromatin, promotes nucleosome remodeling, and enables reactivation of tumor suppressor gene expression

## CDK9 in hematological malignancies

Aberrant overexpression of CDK9 has been observed in various malignancies, where it contributes significantly to tumor growth and serves as a key determinant of prognosis. Its influence is particularly notable in hematologic malignancies, including acute myeloid leukemia (AML) [51] where CDK9 activity contributes to the uncontrolled growth and survival of malignant cells. In acute myeloid leukemia (AML), CDK9 mRNA levels are elevated approximately 2- to 5-fold in leukemic blasts compared with normal myeloid progenitors, where CDK9 activity is essential for sustained transcription of the short-lived anti-apoptotic protein MCL-1 [52]. Similarly, in chronic lymphocytic leukemia (CLL), high CDK9 expression correlates with increased MCL-1 and BCL-2 levels and is associated with poor clinical outcomes [53]. Due to its significant involvement in cancer biology, CDK9 has gained attention as a potential therapeutic target for intervention in various malignancies, among them, chronic lymphatic leukemia [54], B-cell lymphoma [55], adult T-cell leukemia [56], multiple myeloma [MM] [57], and NK-cell leukemia) [58], and solid tumors [2]. A significant number of genes, including c-Myc and Mcl-1, have their expression levels reduced as a consequence of CDK9 inhibition, which finally causes gene transcription to stop as shown in Fig. 1 [59]. In contrast, genetic alterations of CDK9 are uncommon, with gene amplifications occurring in fewer than 5% of hematologic malignancies (compared with up to 15–20% in certain solid tumors such as prostate cancer) [60]. Instead, pathological CDK9 activation predominantly arises from upstream transcriptional and epigenetic dysregulation, including MLL fusion–mediated signaling, aberrant BRD4 recruitment, and disruption of the 7SK snRNP regulatory complex [61]. A wide variety of cellular biological processes, including cell growth, proliferation, differentiation, and apoptosis, are under the control of the oncogene c-Myc [62]. On the other hand, myeloid cell leukemia-1 (MCL1) is a protein that belongs to the BCL2 (B-cell lymphoma 2) family. The downregulation of MCL1 led to the induction of apoptosis, which suggests that it plays a function in the survival of cells [63] Fig 2.

### Leukemia

The disruption of the CDK9 pathway has been reported by numerous studies in acute myeloid leukemia (AML). Higher amounts of cMYC and MCL-1 have been implicated in AML progression by supporting the sustained survival and expansion of these cells [64]. There is an increase in the expression of myeloid cell leukemia 1 (MCL-1), which is necessary for the development and progression of AML [65]. Leukemia cell viability is supported by transient MCL-1 expression, which is continually reinforced by P-TEFb activity [66]. Otherwise, it was found that P-TEFb binds with mixed lineage leukemia (MLL) translocation products in AML, which triggers constitutively active transcription [64]. The levels of P-TEFb were observed to be around twofold in patients with recurrent cases when compared with those prior to treatment, and its reduction significantly increased the mortality of AML cells in human and mouse models [52]. The HOXA gene family, including key regulators like HOXA7 and HOXA9, are upregulated by MLL, which is expressed ubiquitously in myeloid and lymphoid progenitor cells [67, 68]. These genes control hematopoietic stem cell self-renewal and are dramatically downregulated during hematopoietic differentiation [69]. It is estimated that around 10% Within the group of occurrences of AML, regardless of age, are caused by MLL rearrangement leukemia, which has a dismal prognosis. In particular, 35–50% of infant AML and 70–80% of infantile ALL are characterized by MLL fusion [67]. Hence, P-TEFb is extensively recognized for its involvement within the origins of various hematological malignancies, especially leukemia [70]. Blocking CDK9 disrupts the survival mechanism of AML cells by lowering MCL-1 protein levels, which effectively suppresses leukemia progression in animal experiments [71, 72]. Based on these findings, CDK9 inhibitors are now being investigated as part of clinical trials to determine their efficacy for AML treatment [73]. Additionally, pharmacological suppression of CDK9 by flavopiridol and dinaciclib in MLL mouse models, significantly slows disease advancement and improves survival [74–76] Fig 3.

Adult T-cell leukemia/lymphoma (ATL) is a hematological malignancy originating from T-cells [77]. BAY 1,143,572, which successfully inhibited CDK9, resulting in a decrease in RNA polymerase II phosphorylation at serine 2 in cell lines originating from ATL and T-cell lines transformed with HTLV-1 and primary ATL samples. The downregulation of MYC resulted in impaired cellular growth. In addition, lowered MCL1 expression facilitated apoptosis. In immunodeficient mice engrafted with human ATL cells, administration of BAY 1,143,572 led to a marked reduction in ATL cell infiltration into target organs, including the bone marrow and liver. Serum levels of human soluble IL2R were found to be lower, suggesting a decrease in the burden of ATL tumors. In an important research study, Narita, Ishida, and colleagues showed that CDK9-driven transcription is fundamental to the development of ATL. They also proposed that CDK9 inhibitors could be a promising treatment option for ATL [78].

**Fig. 4:**
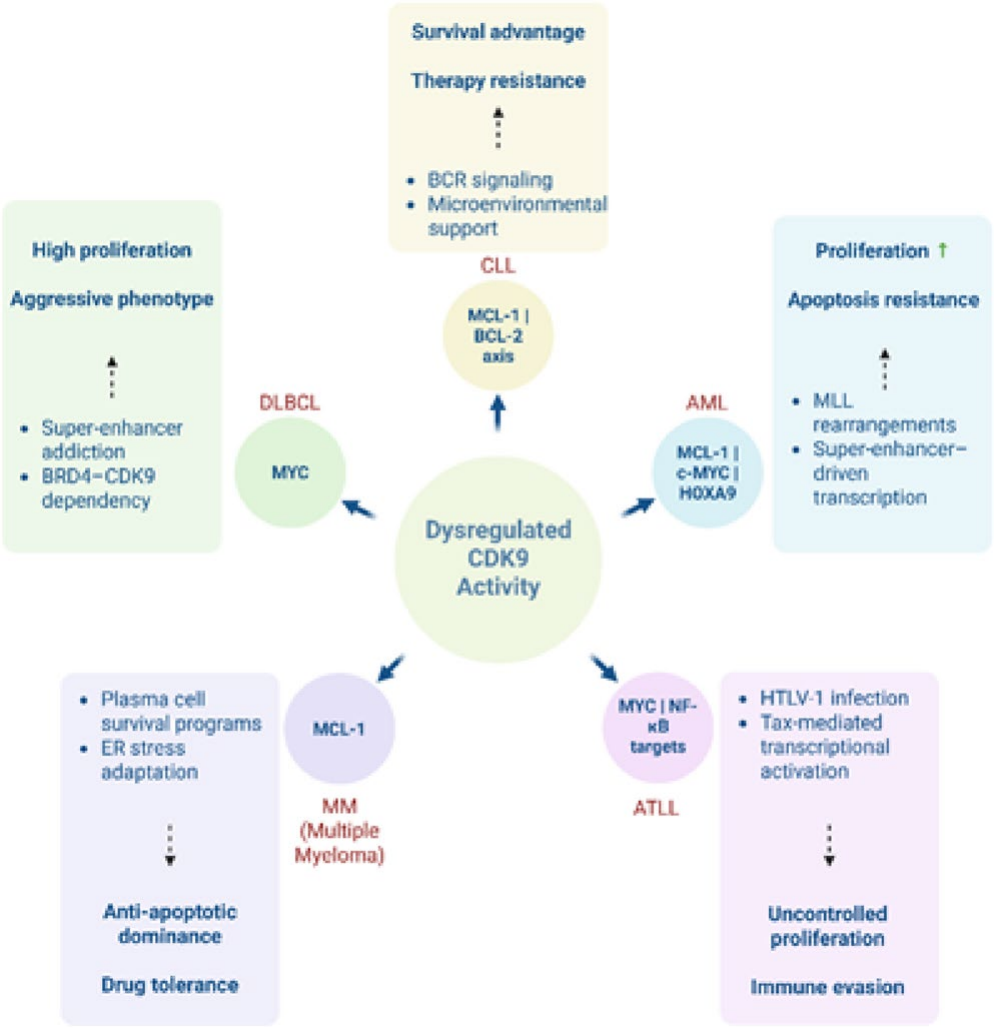
Pathogenic roles of dysregulated CDK9 activity across hematologic malignancies. Aberrant CDK9-driven transcriptional elongation constitutes a shared oncogenic dependency across diverse hematologic cancers. Central dysregulation of CDK9 sustains short-lived oncogenic transcriptional programs, while disease-specific genetic, epigenetic, or viral contexts (e.g., MLL rearrangements in AML, super-enhancer addiction in DLBCL, or HTLV-1 infection in ATLL) shape distinct pathogenic outcomes. This convergence highlights transcriptional addiction as a unifying therapeutic vulnerability

In a research study, inhibition of CDK9 using the selective inhibitor LDC526 caused a significant downregulation of expression MYC and MCL1 in T-cell prolymphocytic leukemia (T-PLL), a rare and highly aggressive T-cell malignancy. Moreover, comprehensive microarray analyses uncovered several novel downstream targets of CDK9, among which the oncoprotein PIM1 stood out. PIM1 has been shown to cooperate closely with MYC in driving oncogenesis and contributes significantly to maintaining cell viability and proliferation in T-cell acute lymphoblastic leukemia (T-ALL). These findings suggest that targeting CDK9 may not only suppress key oncogenic drivers like MYC and MCL1 but also disrupt other survival pathways mediated by proteins such as PIM1, offering a promising therapeutic strategy for aggressive T-cell neoplasms [79] Fig 4.

The most prevalent kind of leukemia in those over the age of 65 is chronic lymphocytic leukemia (CLL), defined by the presence of fully mature B-lymphocytes with impaired function gathering in blood and lymphatic systems [80]. Unlike other malignancies affecting the hematopoietic system, CLL is an indolent form of leukemia characterized by continuous triggering of B-cell receptor signaling and increased expression of apoptosis-suppressing proteins from the B-cell lymphoma 2 family, such as BCL-2, MCL-1 [81, 82]. Studies conducted on CLL cells demonstrated that therapy with flavopiridol results in a decrease in the production of anti-apoptotic proteins such as Bcl2, Mcl-, and XIAP. These proteins are recognized as key contributors to the survival of CLL cells. It is possible that the influence on these genes that promote survival could be mediated by the suppression of P-TEFb and transcription elongation phase [83]. Flavopiridol treatment of CLL patient cells induces autophagy, according to another study. While endoplasmic reticulum (ER) stress in low amounts has been found to protect CLL cells from death, flavopiridol increases ER stress to dangerous levels, activating caspase 4 and apoptosis signal-regulated kinase 1 (ASK1). Consequently, flavopiridol caused more cell death in cells that were previously resistant [84]. Alongside research on flavopiridol as a monotherapy, other studies have examined its use in conjunction with other established drugs to formulate innovative chemotherapeutic protocols for chronic lymphocytic leukemia (CLL) [85]. A novel multimodal medication therapy method was investigated to ascertain whether the A therapeutic approach combining cyclophosphamide, rituximab, and flavopiridol—collectively known as CAR therapy—has demonstrated the ability to lessen severe toxic effects related to tumor lysis syndrome (TLS) in patients with high-risk chronic lymphocytic leukemia (CLL). Notably, clinical studies implementing a modified schedule for flavopiridol administration, where the drug is given later in the treatment cycle, have successfully minimized the incidence of serious TLS complications. These results highlight both the safety and the effectiveness of the CAR regimen in managing aggressive forms of CLL [86].

### Lymphoma

**Fig. 5:**
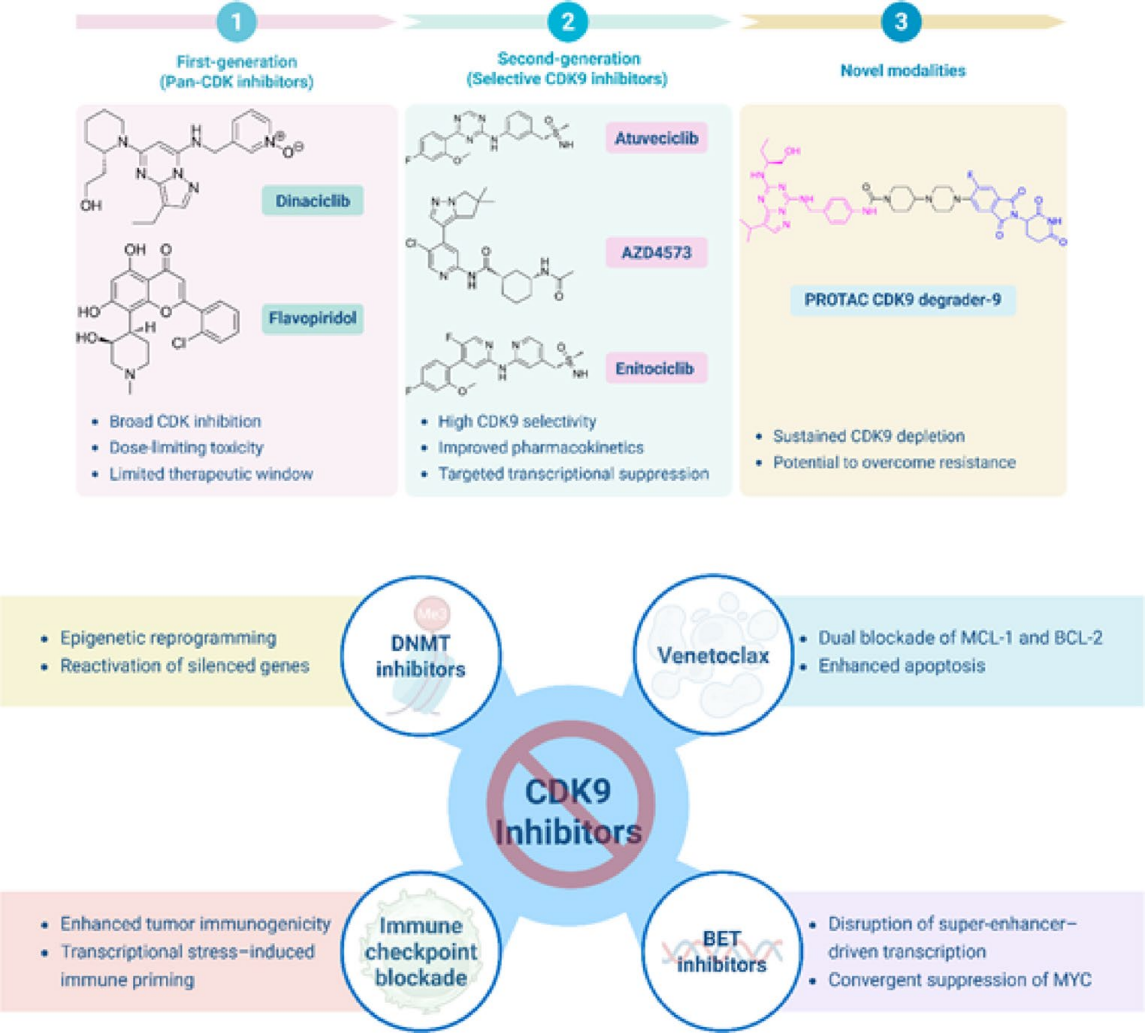
Evolution, mechanisms of action, and rational combination strategies of CDK9-targeted therapies. The development of CDK9-targeted agents has progressed from first-generation pan-CDK inhibitors, such as flavopiridol and dinaciclib, characterized by broad kinase inhibition and limited therapeutic windows, to second-generation, more selective CDK9 inhibitors, including atuveciclib, AZD4573, and VIP152, which demonstrate improved specificity and tolerability. Emerging therapeutic modalities, such as proteolysis-targeting chimeras (PROTACs), further expand this landscape by enabling sustained CDK9 depletion. Owing to its central role in maintaining oncogenic transcriptional programs, CDK9 inhibition provides a rational backbone for combination therapies. Synergistic partners include BCL-2 inhibitors (e.g., venetoclax) to enhance apoptotic priming, epigenetic therapies such as DNMT or BET inhibitors to reinforce transcriptional suppression, and immune checkpoint blockade to potentially augment antitumor immune responses. Collectively, these strategies aim to maximize therapeutic efficacy while overcoming adaptive resistance mechanisms

P-TEFb has been implicated in the pathogenesis of lymphoma, notably diffuse large B-cell lymphoma (DLBCL). Its aberrant activation contributes to dysregulated transcriptional control, promoting uncontrolled proliferation and survival of malignant B cells, thereby facilitating lymphomagenesis [87]. B-cell lymphomas result from the malignant expansion of B cells at multiple stages throughout their developmental journey [88]. The expression of the c-MYC protein is observed in a significant proportion, estimated at around 30–50%, of DLBCL cases [89, 90]. LBCLs exhibiting c-MYC rearrangement demonstrate a heightened propensity for relapse within the central nervous system, irrespective of additional risk factors [91]. The transactivation process mediated by c-Myc relies on P-TEFb, with the activation domain of c-Myc engaging directly with CycT1 [92, 93]. The selective inhibition of CDK9, whether through specific inhibitors such as AZ5576 or via genetic knockdown, has been shown to negatively regulate the expression of MYC and MCL-1, subsequently Triggering apoptotic cell death in both primary and malignant cells originating from this type of lymphoma [55, 94] Fig 5.

### Multiple myeloma

Multiple myeloma (MM) represents the second most prevalent hematological malignancy, distinguished by the proliferation of monoclonal plasma cells, which results in the synthesis of non-functional immunoglobulins [95]. Flavopiridol, known as the first broad-spectrum CDK inhibitor, was shown to decrease the levels of anti-apoptotic proteins in ANBL-6 and ARP1 multiple myeloma cell lines. The decrease in Mcl-1, XIAP, and Bcl-XL levels was linked to the initiation of apoptosis in multiple myeloma cells [96]. Although the initial generation of CDK9 inhibitors proved insufficient as independent treatments, they appear to enhance the effectiveness of other therapeutic agents [97]. Inhibitors that exhibit greater selectivity tend to demonstrate reduced toxicity and are generally well-accepted by patients [61]. AZD-4573, a specifically designed inhibitor of CDK9, has shown efficacy in suppressing the growth of multiple myeloma cell lines in vitro, encompassing those resistant to bortezomib and lenalidomide [98]. In addition, co-treatment with AZD-4573 and ARV825, a PROTAC targeting bromodomain proteins, enhanced the suppression of MCL-1 and Myc levels. This combination therapy induced significantly greater apoptosis in multiple myeloma cell lines than monotherapy [98]. CDK9 inhibitors are presently under extensive examination in multiple myeloma, as discussed in the review by Borowczak et al. [99].

## CDK9 inhibitors as a novel target therapy

CDK9 represents a promising target for cancer therapy. The following sections will discuss emerging strategies for CDK9 inhibition with strong therapeutic potential in hematologic malignancies as shown in Table 1, highlighting ongoing and completed clinical studies involving small-molecule CDK9 inhibitors.

### First generation CDK9 inhibitors

**Fig. 6:**
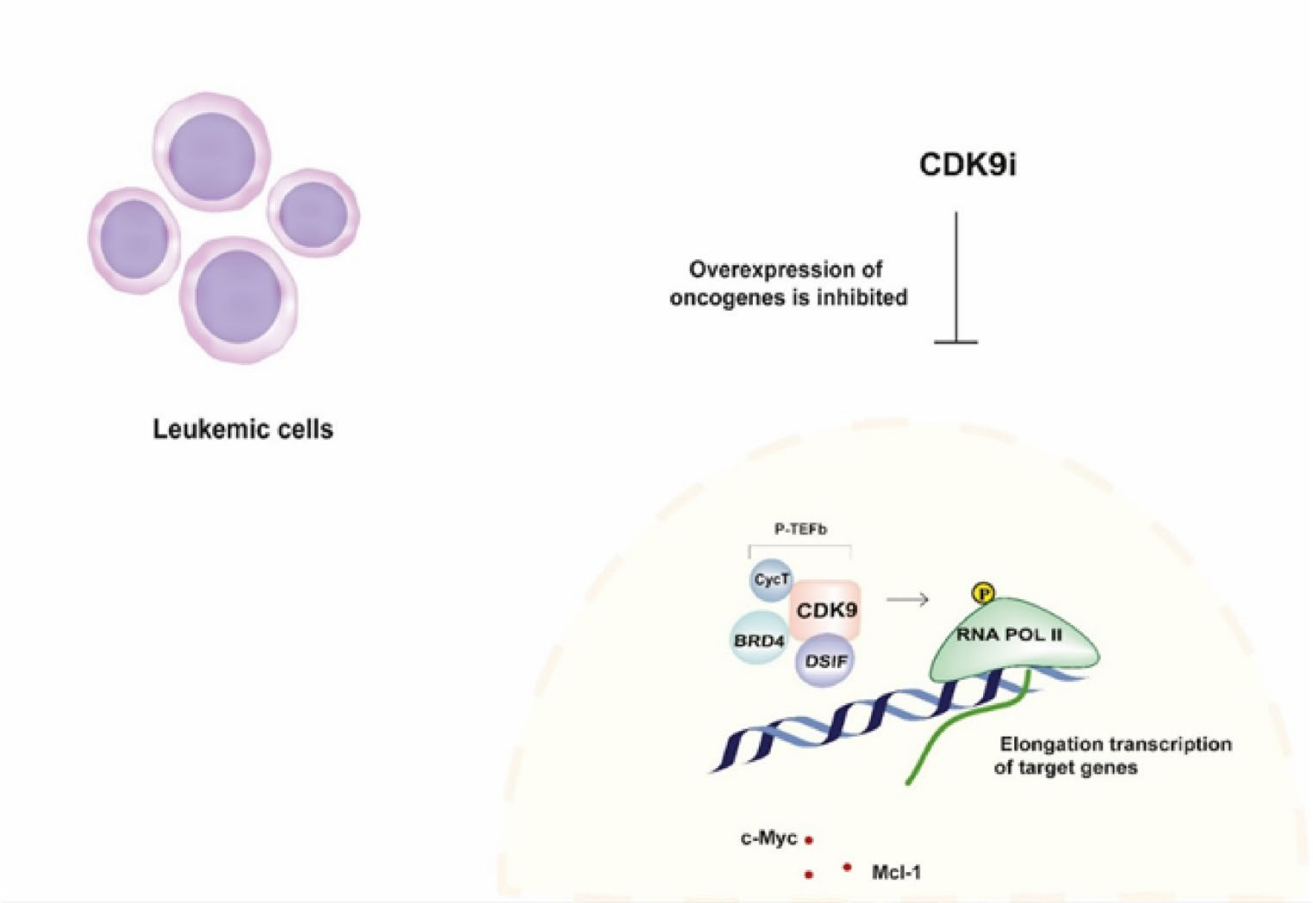
CDK9 localization in the nucleus and its role in promoting transcription of MCL1 and cMYC genes, highlighting its function in gene activation pathways

The initial cohort of inhibitors, such as flavopiridol and seliciclib, frequently referred to as “pan-CDK” inhibitors, engage the ATP-binding site of cyclin-dependent kinases, demonstrating less than optimal clinical therapeutic efficacy [100, 101]. Given the evolutionary conservation of the ATP-binding pocket within the CDKs, early-generation inhibitors exhibited limited specificity for CDK9. Several of these compounds were assessed in clinical trials; however, they were associated with substantial toxicity and demonstrated low complete response rates in patients [102–105]. Flavopiridol functions as an ATP-competitive inhibitor and has exhibited noteworthy in vitro efficacy across various cancer types. However, it’s in vivo effectiveness has been largely restricted, with the exception of hematologic malignancies such as MCL and CLL, ultimately leading to the discontinuation of its further development [10, 106]. Although flavopiridol showed early therapeutic promise, its clinical efficacy was limited compared to its toxicity in trials involving hematologic malignancies, whether administered alone or in combination with other therapeutic agents. However, in contrast, using flavopiridol in carefully timed combination with chemotherapy drugs significantly improved complete remission rates in patients with acute myeloid leukemia, suggesting that scheduling and drug synergy can enhance its clinical effectiveness despite earlier concerns about toxicity [107]. The second pan-CDK inhibitor, seliciclib (also referred to as roscovitine), demonstrated enhanced selectivity compared to flavopiridol. However, it proved less effective in inhibiting CDK9, despite showing greater potency against other CDKs such as CDK2, CDK5, and CDK7. While both inhibitors target a range of cyclin-dependent kinases, seliciclib’s ability to more selectively inhibit certain CDKs makes it a potentially promising candidate for therapies targeting specific cell cycle and transcriptional regulation pathways [108], Seliciclib induced cell cycle arrest and apoptosis, exhibiting significant anticancer effects across a variety of preclinical cancer models [109, 110]. Despite demonstrating anticancer activity, Seliciclib’s application as a monotherapy was limited in clinical settings because of its insufficient effectiveness and considerable adverse effects [111]. Dinaciclib targets several CDKs and, compared to flavopiridol, has demonstrated improved effectiveness and a safer profile in preclinical studies, making it a promising candidate for further development in cancer therapy [112]. It has also been shown to induce apoptosis in CLL patient cells, successfully bypassing the protective effects of cytokines within the tumor microenvironment [113]. Moreover, it exhibits demonstrated strong antiproliferative activity against AML and ALL cell lines in vitro, inducing apoptosis via the intrinsic pathway. This process was associated with the downregulation of Mcl-1, a key anti-apoptotic protein, through the inhibition of CDK9. It also resulted in a reduction of XIAP and Bcl-xL expression, with all findings verified in primary leukemia cells [114]. Dinaciclib was studied in relapsed and refractory chronic lymphocytic leukemia (CLL), resulting in a 54% partial response rate with minimal adverse effects, such as cytopenia and tumor lysis syndrome [115]. Notably, it also facilitates immunogenic cell death while enhancing the efficacy of anti–PD-1 immunotherapy by improving dendritic cell function. These results offer a solid basis for its combination with anti–PD-1 therapy [116]. Further preclinical and clinical investigations are necessary to ascertain the tolerability of this combination and its potential to enhance tumor control Fig 6.

SNS-032 was initially developed as a selective inhibitor of CDK2; however, later studies demonstrated that it also effectively inhibits CDK7 and CDK9, with additional, though less potent, activity against CDKs 1, 4, 5, and 6 [117]. The Phase I trial (NCT00446342) involved 19 patients suffering from CLL and 18 from MM. for the CLL group the maximum tolerated dose could not be established, and dose-limiting toxicity was not observed in the multiple myeloma patients. SNS-032 exhibited commendable tolerance in both patient cohorts across all infusion doses. the reduction in pS2 levels was markedly more pronounced in contrast to pS5 levels. This was coinciding with a decrease in XIAP, MCL-1 levels. Despite its multi-target activity, SNS-032 demonstrated only modest anti-tumor efficacy in clinical trials. In the chronic lymphocytic leukemia (CLL) cohort, one patient achieved a partial response and another exhibited stable disease. Similarly, within the multiple myeloma (MM) cohort, stable disease was observed in one patient. However, treatment discontinuation occurred in all three cases of disease stabilization, caused by toxic effects of the therapy, patient withdrawal, or clinical judgment [118].

### Second generation CDK9 inhibitors

One of the primary limitations in bringing pan-CDK inhibitors into clinical practice is their broad activity, which often results in harmful effects on healthy cells. In response to this challenge, scientists have shifted focus toward designing selective CDK inhibitors—targeting specific kinases like CDK4/6, CDK7, CDK9, and CDK12/13. These more precise agents have shown strong anti-cancer potential in both laboratory studies and clinical settings. This document provides a concise overview of the defining features of select CDK inhibitors. NVP-2, an aminopyrimidine-derived ATP-competitive CDK9 inhibitor characterized by its high selectivity. NVP-2 is a highly potent and selective inhibitor of CDK9. It primarily acts by suppressing CDK9 kinase activity [119]. leading to reduced phosphorylation of RNA polymerase II [120]. This inhibition results in significant antiproliferative effects, primarily through MCL-1 suppression, which in turn triggers apoptotic commitment in malignant cells. Furthermore, NVP-2 has been shown to decrease overall mRNA levels in MOLT4 cells while promoting RNA polymerase II accumulation near promoter regions [119]. In an effort to improve selectivity, the researchers developed a novel strategy by repurposing SNS-032—a broad-spectrum CDK9 inhibitor—through its conjugation to a ligand that engages cereblon (CRBN), an E3 ligase, thereby allowing selective suppression of CDK9 activity. The resulting compound, THAL-SNS-032, not only reduced CDK9 levels but also produced more sustained antiproliferative activity and a stronger apoptotic response.

Atuveciclib (BAY 1143572) demonstrates notable anti-proliferative efficacy against various human cancer cell lines. The impact on CDK9 was over 50 times more significant than that observed with other CDKs [72]. BAY1143572 inhibits phosphorylation of RNA polymerase II at Ser2, leading to the downregulation of anti-apoptotic and proliferation-related genes, thereby inducing apoptosis. currently, it is being assessed in early-phase clinical studies targeting advanced cancer populations [121]. Despite its promising activity, atuveciclib showed limited therapeutic potential in a phase I clinical trial. Targeting MCL-1 in leukocytes led to treatment- related toxicity, with neutropenia emerging as the most common effect. Nevertheless, atuveciclib exhibits potent cytotoxicity against T-PLL cells through the initiation of intrinsic apoptotic signaling pathways. Moreover, its combination with venetoclax produced a synergistic effect, resulting in a more potent leukemia-suppressive response than either agent alone [122]. Additionally, Atuveciclib synergizes with TRAIL to effectively reduce the survival capacity of pancreatic cancer cells, thereby enhancing therapeutic potential [123].

After treating MCF7 cells for a short period of time, Cidado et al. discovered that AZD4573 shows a preference for CDK9 over other CDKs that is more than 25 times higher. These cells had a frameshift mutation in CASP3, which makes them resistant to cell death and apoptosis. AZD4573 induced rapid apoptosis in various cell lines representing hematologic malignancies subjected to short-term exposure (6 h). It was administered biweekly, either as monotherapy or in synergy with venetoclax [124]. AZD4573 demonstrated promising antitumor activity across multiple preclinical models of hematologic malignancies. In several AML patient-derived xenografts, it led to a notable reduction in leukemic burden. It also enhanced cancer cell death in T-cell lymphoma and showed effectiveness in models that were resistant to other treatments. Additionally, AZD4573 induced tumor regression in lymphoma xenografts, supporting its potential as a therapeutic option for aggressive blood cancers [125]. It is now being tested in two Phase I clinical trials for advanced hematological malignancies [126] and refractory or recurrent hematological malignancies (NCT03263637) [127].

MC180295, a selective CDK9 inhibitor, has recently demonstrated potent anticancer activity. Unlike traditional CDK9 inhibitors that primarily act by suppressing transcription elongation, MC180295 appears to exert its antitumor effects through the reactivation of epigenetically silenced tumor suppressor genes, thereby promoting cellular differentiation [49]. Pharmacologic suppression of CDK9 activity led to attenuated phosphorylation of BRG1, highlighting a novel regulatory axis within the SWI/SNF complex and resulting in widespread epigenetic derepression across the genome. This finding suggests that CDK9 may serve as a novel epigenetic regulator. Additionally, CDK9 inhibition triggered an interferon response, activated endogenous retroviruses, and increased the effectiveness of immune checkpoint inhibitors [49]. Moreover, MC180295 does not alter the CD4+/CD8 + T cell ratio in vivo and demonstrates safety toward human T lymphocytes. These findings suggest that MC180295 holds promise as an effective antitumor agent [49]. In a recent study, Zhang et al. explored the therapeutic potential of MC180295 across diverse cancer cell lines and mouse models. The compound exhibited remarkable nanomolar potency, with a striking 20-fold selectivity for CDK9 over other cyclin-dependent kinases. Demonstrating broad-spectrum anticancer activity, MC180295 effectively targeted multiple malignancies, including melanoma, leukemia, and various other tumor types. The compound showed heightened activity against acute myeloid leukemia compared to solid tumors. The data further reveal notable antitumor effects in mouse models, both as a standalone treatment and in combination with DNMT inhibitors. Importantly, its efficacy appears to be partially dependent on the presence and function of T cells [128].

To summarize, CDK9 inhibitors of recent generations are revealing their yet unrealized anti-cancer therapeutic potential, and continued research in this area is paving the way for the development of even more effective and selective inhibitors. It is recommended to take these CDK9 inhibitors in conjunction with other drugs that block specific proteins or complexes of proteins, such as BRD4, SEC, cMYC, HSP90, or MCL-1, delaying or preventing treatment resistance and increasing the CDK9 inhibitors’ efficacy.

### Mechanisms of resistance to CDK9 inhibitors

As part of the P-TEFb complex, CDK9 phosphorylates RNA polymerase II to promote transcription elongation [129]. CDK9 inhibitors disrupt this process, leading to the rapid loss of short-lived oncogenic proteins such as MYC and MCL-1. This loss induces apoptosis in “transcription-addicted” cancers, including hematological malignancies and MYC-dependent tumors. However, resistance to these inhibitors develops through several mechanisms that limit their clinical efficacy [130].

One major mechanism involves point mutations in the kinase domain of CDK9. For example, the L156F mutation alters the ATP-binding pocket and creates steric hindrance, markedly reducing the binding affinity of ATP-competitive inhibitors and even some PROTACs [131]. Although this mutation decreases CDK9 catalytic activity and thermostability, it still permits cell survival. This resistance mechanism has been identified in models of acquired resistance, such as AML cells exposed to BAY1251152, and has been validated by CRISPR-based studies, kinase assays, and xenograft experiments [132].

Another important mechanism is the paradoxical and compensatory upregulation of MYC. Prolonged CDK9 inhibition disrupts the inhibitory 7SK snRNP complex, resulting in the release of P-TEFb. BRD4 then recruits P-TEFb to chromatin, particularly at the MYC locus [133]. Consequently, MYC transcription is restored despite a global impairment of elongation, and survival-associated genes are re-expressed. This phenomenon has been observed with selective inhibitors such as i-CDK9 and is dependent on BRD4 activity [134]. Concurrent BRD4 inhibition, for example with JQ1, can suppress this effect [135].

Epigenetic reprogramming and super-enhancer restoration also contribute to resistance. Although CDK9 inhibition initially alters chromatin accessibility, super-enhancers can sustain or re-establish oncogene expression, including MYC and PIM3. This process involves components of the Mediator complex, such as MED12, and signaling pathways including PI3K/AKT. Such effects have been reported in lymphoma models treated with AZD4573, where MED12 knockout enhances drug sensitivity [136].

Finally, activation of compensatory signaling pathways, such as ERK–MYC or HSP90–MYC–CDK9 axes, helps maintain MYC stability or engages alternative transcription factors. Upregulation of anti-apoptotic proteins, including MCL-1, may also occur through feedback mechanisms or the activity of other CDKs [137].

To overcome these resistance mechanisms, several strategies have been proposed. These include CDK9 degraders (PROTACs) that rapidly eliminate the CDK9 protein and limit paradoxical MYC rebound and compensatory feedback; combination therapies targeting BRD4, PI3K/AKT, PIM kinases, or MYC itself; and the development of novel inhibitors, such as IHMT-CDK9-36, that retain activity against L156F-mutant CDK9 Fig 4.

### Current consensus on monotherapy vs. combination therapy

Although the manuscript primarily discusses CDK9 inhibitors in the context of monotherapy, reviews regarding combination therapies with CDK9 inhibitors in hematologic malignancies have also been published. Emerging evidence strongly supports the superiority of combination approaches over monotherapy in most clinical scenarios. CDK9 inhibitors have shown promising activity as single agents in preclinical models and early-phase trials, particularly in transcriptionally addicted cancers reliant on short-lived proteins such as MYC and MCL-1. However, monotherapy often results in transient responses due to the rapid development of resistance mechanisms, including compensatory MYC upregulation, epigenetic reprogramming, and activation of alternative survival pathways.

Recent comprehensive reviews emphasize that CDK9 inhibitors serve as highly effective combination partners in hematologic malignancies. For example, combinations with BCL-2 inhibitors (e.g., venetoclax) demonstrate profound synergy in AML, CLL, and lymphoma models by simultaneously disrupting transcriptional elongation and mitochondrial apoptosis pathways, leading to deeper and more durable remissions [138]. Similarly, pairing CDK9 inhibitors with BTK inhibitors (e.g., ibrutinib) or PI3K inhibitors overcomes microenvironmental protection and resistance in B-cell malignancies [139]. Clinical data, such as the combination of the oral CDK9 inhibitor Voruciclib with venetoclax in relapsed/refractory AML, have shown objective responses in heavily pretreated patients, with on-target pharmacodynamic effects and manageable toxicity [140]. The current consensus in the field is that combination therapy is the more effective approach for CDK9 inhibitors in hematologic malignancies. Rational combinations not only enhance antitumor efficacy but also mitigate resistance, allow for dose reduction to improve tolerability, and broaden the therapeutic window. Monotherapy may be suitable for select patients with high transcriptional dependency and minimal resistance mechanisms, but combination regimens represent the standard for optimal outcomes and are the focus of ongoing and future clinical development Figs 5 and 6

## Conclusion

Taken together, the evidence positions CDK9 (through its core function in the P-TEFb complex with cyclin T1) as a master regulator of transcriptional elongation and a critical driver of transcriptional addiction in hematologic malignancies. Its aberrant activity sustains the expression of short-lived oncogenic drivers and anti-apoptotic proteins, generating a selective vulnerability in malignant cells relative to normal counterparts. Targeted CDK9 inhibition thus emerges as a compelling therapeutic strategy with the potential for an improved therapeutic index. Preclinical studies have validated potent antitumor effects with selective CDK9 inhibitors, including AZD4573, VIP152 (formerly known as enitociclib in some contexts) [141], PRT2527 [142], and compounds from diverse structural classes, particularly in blood cancers. Several of these agents have entered early-phase clinical trials, demonstrating translational promise. While insights into mechanisms such as MCL-1 downregulation have largely stemmed from less selective inhibitors, newer highly selective tools are enabling deeper exploration of CDK9-specific effects beyond established targets like MCL-1 and MYC. Combination approaches further amplify therapeutic potential, with preclinical synergy observed alongside BCL-2 inhibitors (e.g., venetoclax) [143], DNA-damaging agents (e.g., temozolomide, cisplatin) [144], apoptosis inducers (e.g., TRAIL) [145], MDM2 antagonists (e.g., nutlin-3a) [146], and BET inhibitors (e.g., JQ1) [135]. Defining optimal regimens remains a priority. Despite this progress, key translational challenges must be addressed to realize clinical impact. First, achieving a favorable therapeutic index requires balancing potent antitumor activity against on-target toxicities in normal proliferating tissues, where CDK9 supports basal transcription. Second, robust pharmacodynamic biomarkers, such as phosphorylation of RNA polymerase II at Ser2 (p-RNAPII Ser2), are essential for guiding dosing schedules and monitoring target engagement. Third, patient stratification demands molecular signatures, including transcriptional dependency scores, to identify likely responders and exclude non-responders. Moving forward, systems-level analyses (e.g., transcriptomic and epigenomic profiling) are critically needed to delineate the full consequences of CDK9 inhibition, uncover novel biomarkers, and reveal synthetic lethal partners beyond current paradigms. Emerging modalities, such as proteolysis-targeting chimeras (PROTACs) designed to degrade CDK9 (e.g., dCDK9-202 and related degraders) [147], offer potential advantages in selectivity, depth of target suppression, and overcoming resistance mechanisms associated with catalytic inhibitors Table 2.

In conclusion, selective CDK9 inhibition holds transformative potential for hematologic malignancies, provided ongoing efforts surmount these hurdles through refined inhibitors, rational combinations, and precision-guided strategies. As multimodal interrogations deepen our understanding of transcriptional dysregulation in cancer, CDK9 targeting may not only yield effective therapies but also illuminate broader epigenetic and immune-modulatory networks.

**Table 1:** Clinical trials of CDK9 inhibitors in hematologic malignancies

Drug	Neoplasm	Status	Phase	clinicaltrial.gov
**Dinacilib**	Chronic Lymphocytic Leukemia	Completed	ꞮꞮꞮ	NCT01580228
	multiple myeloma	Completed	ꞮꞮ	NCT01096342
	Triple Negative Breast Cancer	Completed	Ɪ	NCT01676753
	Acute Myeloid Leukemia	Terminated	Ɪ	NCT03484520
	Malignant Solid Neoplasm	Active, not recruiting	Ɪ	NCT01434316
	Multiple cancer entities	Active, not recruiting	ꞮꞮ	NCT00937937
**Dinaciclib/SCH-727,965**	Acute Myelogenous Leukemia and Acute Lymphoblastic Leukemia	Terminated	ꞮꞮ	NCT00798213
	Solid Tumors, Non-Hodgkin Lymphoma, Multiple Myeloma, and Chronic Lymphocytic Leukemia	Completed	ꞮꞮ	NCT00871663
	Chronic Lymphocytic Leukemia and Small Lymphocytic Lymphoma	Completed	Ɪ	NCT01650727
	Breast Neoplasms and Non-Small-Cell Lung Cancer	Completed	ꞮꞮ	NCT00732810
**AT-7519**	Chronic Lymphocytic Leukemia	Completed	ꞮꞮ	NCT01627054
	Advanced/metastatic/unresectable solid Neoplasms	Active, not recruiting	Ɪ	NCT02503709
	Lymphoma	Completed	Ɪ	NCT00390117
	Mantle Cell Lymphoma	Completed	ꞮꞮ	NCT01652144
**AT-7519 + Bortezomib**	multiple myeloma	Completed	Ɪ/ꞮꞮ	NCT01183949
**Alvocidib/Flavopiridol**	Chronic Lymphocytic Leukemia, Prolymphocytic Leukemia arising from Chronic Lymphocytic Leukemia	Completed	ꞮꞮ	NCT00464633
	Ovarian Epithelial Cancer and Primary Peritoneal Cancer	Completed	ꞮꞮ	NCT00083122
	Acute Myeloid Leukemia	Completed	ꞮꞮ	NCT00407966
	Myelodysplastic Syndromes	Terminated	Ɪ/ꞮꞮ	NCT03593915
	Lymphoma and Multiple Myeloma	Terminated	Ɪ/ꞮꞮ	NCT00112723
	Unspecified Adult Solid Tumor, Protocol Specific	Terminated	Ɪ	NCT00112684
	B-Cell Neoplasms	Completed	Ɪ	NCT00082784
	Relapsed or Refractory Acute Leukemia	Completed	Ɪ	NCT00470197
**Atuveciclib**	Acute Leukemia	Completed	Ɪ	NCT02345382
**P276-00**	Mantle Cell Lymphoma	Terminated	ꞮꞮ	NCT00843050
	multiple myeloma	Completed	Ɪ/ꞮꞮ	NCT00882063
**Riviciclib/P-276-00**	Squamous Cell Carcinoma of Head and Neck	Completed	ꞮꞮ	NCT00824343
	Pancreatic Cancer	Completed	Ɪ/ꞮꞮ	NCT00898287
	Squamous Cell Carcinoma of Head and neck	Completed	Ɪ/ꞮꞮ	NCT00899054
	Breast Cancer	Terminated	Ɪ	NCT01333137
**Roniciclib/BAY-**	Solid Tumors	Withdrawn	ꞮꞮ	NCT02656849
**1,000,394**	Small Cell Lung Carcinoma	Terminated	ꞮꞮ	NCT02161419
	Non-Small Cell Lung Cancer	Withdrawn	Ɪ/ꞮꞮ	NCT02522910
	Small Cell Lung Cancer	Terminated	Ɪ/ꞮꞮ	NCT01573338
	Neoplasms	Completed	Ɪ	NCT01188252
**TP-1287**	Advanced Solid Tumors	Terminated	Ɪ	NCT03604783
**Zotiraciclib/TG02/SB1317**	Recurrent Anaplastic Astrocytoma and Glioblastoma	Completed	Ɪ/ꞮꞮ	NCT02942264
	Anaplastic Astrocytoma and Glioblastoma	Completed	Ɪ	NCT03224104
	AML, ALL, Blast Crisis, MDS, Multiple Myeloma	Completed	Ɪ	NCT01204164
	Hepatocellular Carcinoma	Withdrawn	Ɪ	NCT03738111
	Rectal Cancer	Terminated	Ɪ	NCT02933944
	Chronic Lymphocytic Leukemia and Small Lymphocytic Lymphoma	Completed	Ɪ	NCT01699152
**CYC065**	Solid tumors or lymphomas	Completed	Ɪ	NCT02552953
	Acute Myeloid Leukemia and Myelodysplastic Syndromes	Completed	Ɪ	NCT04017546:
**CYC065/Fadraciclib**	Refractory Chronic Lymphocytic Leukemia	Completed	Ɪ	NCT03739554
**CYC-202 (Roscovitine)**	Triple Negative Breast Cancer	Withdrawn	Ɪ	NCT01333423
	Non-small Cell Lung Cancer	Terminated	ꞮꞮ	NCT00372073
	Solid Tumors	Completed	Ɪ	NCT00999401
	Cushing Disease	Terminated	ꞮꞮ	NCT02160730
		Recruiting	ꞮꞮ	NCT03774446
**AZD-4573**	Hematological malignancies	Active, not recruiting	Ɪ/ꞮꞮ	NCT04630756
	multiple myeloma	Completed	Ɪ	NCT03263637
**BAY-1,251,152**	Hematological malignancies	Completed	Ɪ	NCT02745743
	Neoplasms	Completed	Ɪ	NCT02635672
**Voruciclib**	Hematological malignancies	Recruiting	Ɪ	NCT03547115
**GFH009**	Hematological malignancies	Recruiting	Ɪ/ꞮꞮ	NCT04588922
**KB-0742**	Relapsed or Refractory Solid Tumors and Non-Hodgkin’s Lymphoma	Active, not recruiting	Ɪ/ꞮꞮ	NCT04718675
**NMS-1,116,354**	Advanced Solid Tumors	Terminated (Discontinuation of clinical investigation of drug)	Ɪ	NCT01016327
	Advanced/Metastatic Solid Tumors	Terminated (Discontinuation of clinical investigation of drug)	Ɪ	NCT01092052
**SNS-032**	multiple myeloma	Completed	Ɪ	NCT00446342
	solid tumors	Completed	Ɪ	NCT00292864
**RGB-286,638**	multiple myeloma	Withdrawn (sponsor decision)	Ɪ	NCT01168882
**Flavopiridol (Alvocidib)**	Advanced Solid Tumors	Terminated	Ɪ	NCT03604783
	Acute Myeloid Leukemia	Terminated	ꞮꞮ	NCT03969420
	Myelodysplastic Syndromes	Terminated	Ɪ/ꞮꞮ	NCT03593915
**BAY-1,143,572 (Atuveciclib)**	Acute leukaemia	Completed	Ɪ	NCT02345382
	Neoplasms	Completed	Ɪ	NCT01938638
**BTX-A51**	Acute Myeloid Leukemia Myelodysplastic Syndrome	Recruiting	Ɪ	NCT04243785

**Table 2:** Comparison of 1st vs. 2nd generation CDK9 inhibitors and activity in hematologic vs. solid tumors

Generation	Examples	Selectivity (CDK9 vs. others)	Key Toxicity	Efficacy in Hematologic Malignancies	Efficacy in Solid Tumors	Status
1st (pan)	Flavopiridol, Dinaciclib	Low	High (TLS, neutropenia)	Modest as single-agent; improved in combination regimens	Limited	Many programs terminated
2nd (selective)	AZD4573, BAY1251152, Voruciclib, GFH009, PRT2527	High	Manageable neutropenia	Potent apoptosis (MCL-1↓) with high Overall Response Rate	Promising in MYC-amplified tumors	Ongoing Phase I/II

## Data Availability

Data supporting the findings in this study are immediately available upon reasonable request.

## References

[1] Zhou F, Tang L, Le S, Ge M, Cicic D, Xie F, et al. The pharmacodynamic and mechanistic foundation for the antineoplastic effects of GFH009, a potent and highly selective CDK9 inhibitor for the treatment of hematologic malignancies. Oncotarget. 2023;14:997–1008. 38117531 10.18632/oncotarget.28543 PMC10732257

[2] Mo C, Wei N, Li T, Ahmed Bhat M, Mohammadi M, Kuang C. CDK9 inhibitors for the treatment of solid tumors. Biochem Pharmacol. 2024;229:116470. 39127153 10.1016/j.bcp.2024.116470 PMC11580798

[3] Arellano M, Moreno S. Regulation of CDK/cyclin complexes during the cell cycle. Int J Biochem Cell Biol. 1997;29(4):559–73. 9363633 10.1016/s1357-2725(96)00178-1

[4] Swaffer MP, Jones AW, Flynn HR, Snijders AP, Nurse P. CDK substrate phosphorylation and ordering the cell cycle. Cell. 2016;167(7):1750–61. e16. 27984725 10.1016/j.cell.2016.11.034 PMC5161751

[5] Lim S, Kaldis P. Cdks, cyclins and CKIs: roles beyond cell cycle regulation. Development. 2013;140(15):3079–93. 23861057 10.1242/dev.091744

[6] Asghar U, Witkiewicz AK, Turner NC, Knudsen ES. The history and future of targeting cyclin-dependent kinases in cancer therapy. Nat Rev Drug Discovery. 2015;14(2):130–46. 25633797 10.1038/nrd4504 PMC4480421

[7] Węsierska-Gądek J, Maurer M, Zulehner N, Komina O. Whether to target single or multiple CDKs for therapy? That is the question. J Cell Physiol. 2011;226(2):341–9. 20836132 10.1002/jcp.22426

[8] Hirose Y, Ohkuma Y. Phosphorylation of the C-terminal domain of RNA polymerase II plays central roles in the integrated events of eucaryotic gene expression. J Biochem. 2007;141(5):601–8. 17405796 10.1093/jb/mvm090

[9] Koumenis C, Giaccia A. Transformed cells require continuous activity of RNA polymerase II to resist oncogene-induced apoptosis. Mol Cell Biol. 1997;17(12):7306–16. 9372962 10.1128/mcb.17.12.7306 PMC232587

[10] Chou J, Quigley DA, Robinson TM, Feng FY, Ashworth A. Transcription-Associated Cyclin-Dependent Kinases as Targets and Biomarkers for Cancer Therapy. Cancer Discov. 2020;10(3):351–70. 32071145 10.1158/2159-8290.CD-19-0528

[11] Bradner JE, Hnisz D, Young RA. Transcriptional Addiction in Cancer. Cell. 2017;168(4):629–43. 28187285 10.1016/j.cell.2016.12.013 PMC5308559

[12] Kwiatkowski N, Zhang T, Rahl PB, Abraham BJ, Reddy J, Ficarro SB, et al. Targeting transcription regulation in cancer with a covalent CDK7 inhibitor. Nature. 2014;511(7511):616–20. 25043025 10.1038/nature13393 PMC4244910

[13] Lovén J, Hoke HA, Lin CY, Lau A, Orlando DA, Vakoc CR, et al. Selective inhibition of tumor oncogenes by disruption of super-enhancers. Cell. 2013;153(2):320–34. 23582323 10.1016/j.cell.2013.03.036 PMC3760967

[14] Hnisz D, Abraham BJ, Lee TI, Lau A, Saint-André V, Sigova AA, et al. Super-enhancers in the control of cell identity and disease. Cell. 2013;155(4):934–47. 24119843 10.1016/j.cell.2013.09.053 PMC3841062

[15] Whyte WA, Orlando DA, Hnisz D, Abraham BJ, Lin CY, Kagey MH, et al. Master transcription factors and mediator establish super-enhancers at key cell identity genes. Cell. 2013;153(2):307–19. 23582322 10.1016/j.cell.2013.03.035 PMC3653129

[16] Huang CH, Lujambio A, Zuber J, Tschaharganeh DF, Doran MG, Evans MJ, et al. CDK9-mediated transcription elongation is required for MYC addiction in hepatocellular carcinoma. Genes Dev. 2014;28(16):1800–14. 25128497 10.1101/gad.244368.114 PMC4197965

[17] Gregory GP, Hogg SJ, Kats LM, Vidacs E, Baker AJ, Gilan O, et al. CDK9 inhibition by dinaciclib potently suppresses Mcl-1 to induce durable apoptotic responses in aggressive MYC-driven B-cell lymphoma in vivo. Leukemia. 2015;29(6):1437–41. 25578475 10.1038/leu.2015.10 PMC4498453

[18] Baumli S, Lolli G, Lowe ED, Troiani S, Rusconi L, Bullock AN, et al. The structure of P-TEFb (CDK9/cyclin T1), its complex with flavopiridol and regulation by phosphorylation. Embo j. 2008;27(13):1907–18. 18566585 10.1038/emboj.2008.121 PMC2486423

[19] Hanks SK, Hunter T. The eukaryotic protein kinase superfamily: kinase (catalytic) domain structure and classification. FASEB J. 1995;9(8):576–96. 7768349

[20] De Bondt HL, Rosenblatt J, Jancarik J, Jones HD, Morgan DO, Kim S-H. Crystal structure of cyclin-dependent kinase 2. Nature. 1993;363(6430):595–602. 8510751 10.1038/363595a0

[21] Morales F, Giordano A. Overview of CDK9 as a target in cancer research. Cell Cycle. 2016;15(4):519–27. 26766294 10.1080/15384101.2016.1138186 PMC5056610

[22] Shore SM, Byers SA, Maury W, Price DH. Identification of a novel isoform of Cdk9. Gene. 2003;307:175–82. 12706900 10.1016/s0378-1119(03)00466-9

[23] Liu H, Herrmann CH. Differential localization and expression of the Cdk9 42k and 55k isoforms. J Cell Physiol. 2005;203(1):251–60. 15452830 10.1002/jcp.20224

[24] Sung TL, Rice AP. Effects of prostratin on Cyclin T1/P-TEFb function and the gene expression profile in primary resting CD4 + T cells. Retrovirology. 2006;3:66. 17014716 10.1186/1742-4690-3-66 PMC1599745

[25] Rodríguez-Molina JB, West S, Passmore LA. Knowing when to stop: Transcription termination on protein-coding genes by eukaryotic RNAPII. Mol Cell. 2023;83(3):404–15. 36634677 10.1016/j.molcel.2022.12.021 PMC7614299

[26] Cramer P. Eukaryotic Transcription Turns 50. Cell. 2019;179(4):808–12. 31675494 10.1016/j.cell.2019.09.018

[27] Grünberg S, Hahn S. Structural insights into transcription initiation by RNA polymerase II. Trends Biochem Sci. 2013;38(12):603–11. 24120742 10.1016/j.tibs.2013.09.002 PMC3843768

[28] Chen FX, Smith ER, Shilatifard A. Born to run: control of transcription elongation by RNA polymerase II. Nat Rev Mol Cell Biol. 2018;19(7):464–78. 29740129 10.1038/s41580-018-0010-5

[29] Corden JL. RNA polymerase II C-terminal domain: Tethering transcription to transcript and template. Chem Rev. 2013;113(11):8423–55. 24040939 10.1021/cr400158h PMC3988834

[30] Komarnitsky P, Cho EJ, Buratowski S. Different phosphorylated forms of RNA polymerase II and associated mRNA processing factors during transcription. Genes Dev. 2000;14(19):2452–60. 11018013 10.1101/gad.824700 PMC316976

[31] Rimel JK, Taatjes DJ. The essential and multifunctional TFIIH complex. Protein Sci. 2018;27(6):1018–37. 29664212 10.1002/pro.3424 PMC5980561

[32] Zhou Q, Li T, Price DH. RNA polymerase II elongation control. Annu Rev Biochem. 2012;81:119–43. 22404626 10.1146/annurev-biochem-052610-095910 PMC4273853

[33] Coin F, Egly JM. Revisiting the Function of CDK7 in Transcription by Virtue of a Recently Described TFIIH Kinase Inhibitor. Mol Cell. 2015;59(4):513–4. 26295956 10.1016/j.molcel.2015.08.006

[34] Yang Z, Yik JH, Chen R, He N, Jang MK, Ozato K, et al. Recruitment of P-TEFb for stimulation of transcriptional elongation by the bromodomain protein Brd4. Mol Cell. 2005;19(4):535–45. 16109377 10.1016/j.molcel.2005.06.029

[35] Fujinaga K. P-TEFb as A Promising Therapeutic Target. Molecules. 2020;25(4):838. 10.3390/molecules25040838 PMC7070488 32075058

[36] Peterlin BM, Price DH. Controlling the elongation phase of transcription with P-TEFb. Mol Cell. 2006;23(3):297–305. 16885020 10.1016/j.molcel.2006.06.014

[37] Guenther MG, Levine SS, Boyer LA, Jaenisch R, Young RA. A chromatin landmark and transcription initiation at most promoters in human cells. Cell. 2007;130(1):77–88. 17632057 10.1016/j.cell.2007.05.042 PMC3200295

[38] Muse GW, Gilchrist DA, Nechaev S, Shah R, Parker JS, Grissom SF, et al. RNA polymerase is poised for activation across the genome. Nat Genet. 2007;39(12):1507–11. 17994021 10.1038/ng.2007.21 PMC2365887

[39] Eifler TT, Shao W, Bartholomeeusen K, Fujinaga K, Jäger S, Johnson JR, et al. Cyclin-dependent kinase 12 increases 3’ end processing of growth factor-induced c-FOS transcripts. Mol Cell Biol. 2015;35(2):468–78. 25384976 10.1128/MCB.01157-14 PMC4272423

[40] Yu M, Yang W, Ni T, Tang Z, Nakadai T, Zhu J, et al. RNA polymerase II-associated factor 1 regulates the release and phosphorylation of paused RNA polymerase II. Science. 2015;350(6266):1383–6. 26659056 10.1126/science.aad2338 PMC8729149

[41] Wong RWJ, Ishida T, Sanda T. Targeting General Transcriptional Machinery as a Therapeutic Strategy for Adult T-Cell Leukemia. Molecules. 2018;23(5):1057. 10.3390/molecules23051057 PMC6099935 29724031

[42] Cerami E, Gao J, Dogrusoz U, Gross BE, Sumer SO, Aksoy BA, et al. The cBio cancer genomics portal: an open platform for exploring multidimensional cancer genomics data. Cancer Discov. 2012;2(5):401–4. 22588877 10.1158/2159-8290.CD-12-0095 PMC3956037

[43] Puidebat O, Egloff S. The 7SK snRNP complex: a critical regulator in carcinogenesis. Biochimie. 2025;238:3–8. 40368082 10.1016/j.biochi.2025.05.003

[44] Yang Y, Murrali MG, Galvan S, Wang Y, Stephen C, Ajjampore N, et al. HEXIM1 inter-monomer autoinhibition governs 7SK RNA binding specificity and P-TEFb inactivation. Nat Commun. 2026;17(1):1570. 41540012 10.1038/s41467-026-68285-8 PMC12901311

[45] Kotekar A, Singh AK, Devaiah BN. BRD4 and MYC: power couple in transcription and disease. Febs j. 2023;290(20):4820–42. 35866356 10.1111/febs.16580 PMC9867786

[46] Moreno RY, Juetten KJ, Panina SB, Butalewicz JP, Floyd BM, Venkat Ramani MK, et al. Distinctive interactomes of RNA polymerase II phosphorylation during different stages of transcription. iScience. 2023;26(9):107581. 37664589 10.1016/j.isci.2023.107581 PMC10470302

[47] Fujinaga K, Huang F, Peterlin BM. P-TEFb: The master regulator of transcription elongation. Mol Cell. 2023;83(3):393–403. 36599353 10.1016/j.molcel.2022.12.006 PMC9898187

[48] Pan L, Xie W, Li KL, Yang Z, Xu J, Zhang W, et al. Heterochromatin remodeling by CDK12 contributes to learning in Drosophila. Proc Natl Acad Sci U S A. 2015;112(45):13988–93. 26508632 10.1073/pnas.1502943112 PMC4653196

[49] Zhang H, Pandey S, Travers M, Sun H, Morton G, Madzo J, et al. Targeting CDK9 Reactivates Epigenetically Silenced Genes in Cancer. Cell. 2018;175(5):1244–e5826. 30454645 10.1016/j.cell.2018.09.051 PMC6247954

[50] Fardi M, Solali S, Farshdousti Hagh M. Epigenetic mechanisms as a new approach in cancer treatment: An updated review. Genes Dis. 2018;5(4):304–11. 30591931 10.1016/j.gendis.2018.06.003 PMC6303480

[51] Beauchamp EM, Abedin SM, Radecki SG, Fischietti M, Arslan AD, Blyth GT, et al. Identification and targeting of novel CDK9 complexes in acute myeloid leukemia. Blood J Am Soc Hematol. 2019;133(11):1171–85. 10.1182/blood-2018-08-870089 PMC6418475 30587525

[52] Lee DJ, Zeidner JF. Cyclin-dependent kinase (CDK) 9 and 4/6 inhibitors in acute myeloid leukemia (AML): a promising therapeutic approach. Expert Opin Investig Drugs. 2019;28(11):989–1001. 31612739 10.1080/13543784.2019.1678583

[53] Göthert JR, Imsak R, Möllmann M, Kesper S, Göbel M, Dührsen U, et al. Potent anti-leukemic activity of a specific cyclin-dependent kinase 9 inhibitor in mouse models of chronic lymphocytic leukemia. Oncotarget. 2018;9(41):26353–69. 29899864 10.18632/oncotarget.25293 PMC5995184

[54] Chen R, Keating MJ, Gandhi V, Plunkett W. Transcription inhibition by flavopiridol: mechanism of chronic lymphocytic leukemia cell death. Blood. 2005;106(7):2513–9. 15972445 10.1182/blood-2005-04-1678 PMC1895272

[55] Hashiguchi T, Bruss N, Best S, Lam V, Danilova O, Paiva CJ, et al. Cyclin-dependent kinase-9 is a therapeutic target in MYC-expressing diffuse large B-cell lymphoma. Mol Cancer Ther. 2019;18(9):1520–32. 31243099 10.1158/1535-7163.MCT-18-1023

[56] Narita T, Ishida T, Ito A, Masaki A, Kinoshita S, Suzuki S, et al. Cyclin-dependent kinase 9 is a novel specific molecular target in adult T-cell leukemia/lymphoma. Blood J Am Soc Hematol. 2017;130(9):1114–24. 10.1182/blood-2016-09-741983 28646117

[57] Manohar SM, Rathos MJ, Sonawane V, Rao SV, Joshi KS. Cyclin-dependent kinase inhibitor, P276-00 induces apoptosis in multiple myeloma cells by inhibition of Cdk9-T1 and RNA polymerase II-dependent transcription. Leuk Res. 2011;35(6):821–30. 21216463 10.1016/j.leukres.2010.12.010

[58] Kinoshita S, Ishida T, Ito A, Narita T, Masaki A, Suzuki S, et al. Cyclin-dependent kinase 9 as a potential specific molecular target in NK-cell leukemia/lymphoma. Haematologica. 2018;103(12):2059. 30076184 10.3324/haematol.2018.191395 PMC6269314

[59] Boffo S, Damato A, Alfano L, Giordano A. CDK9 inhibitors in acute myeloid leukemia. J Exp Clin Cancer Res. 2018;37(1):36. 29471852 10.1186/s13046-018-0704-8 PMC5824552

[60] Rahman R, Rahaman MH, Hanson AR, Choo N, Xie J, Townley SL, et al. CDK9 inhibition constrains multiple oncogenic transcriptional and epigenetic pathways in prostate cancer. Br J Cancer. 2024;131(6):1092–105. 39117800 10.1038/s41416-024-02810-8 PMC11405875

[61] Anshabo AT, Milne R, Wang S, Albrecht H. CDK9: A Comprehensive Review of Its Biology, and Its Role as a Potential Target for Anti-Cancer Agents. Front Oncol. 2021;11:678559. 34041038 10.3389/fonc.2021.678559 PMC8143439

[62] García-Gutiérrez L, Delgado MD, León J. MYC Oncogene Contributions to Release of Cell Cycle Brakes. Genes (Basel). 2019;10(3):244. 10.3390/genes10030244 PMC6470592 30909496

[63] Krajewski S, Bodrug S, Krajewska M, Shabaik A, Gascoyne R, Berean K, et al. Immunohistochemical analysis of Mcl-1 protein in human tissues. Differential regulation of Mcl-1 and Bcl-2 protein production suggests a unique role for Mcl-1 in control of programmed cell death in vivo. Am J Pathol. 1995;146(6):1309. 7778670 PMC1870904

[64] Franco LC, Morales F, Boffo S, Giordano A. CDK9: A key player in cancer and other diseases. J Cell Biochem. 2018;119(2):1273–84. 28722178 10.1002/jcb.26293

[65] Glaser SP, Lee EF, Trounson E, Bouillet P, Wei A, Fairlie WD, et al. Anti-apoptotic Mcl-1 is essential for the development and sustained growth of acute myeloid leukemia. Genes Dev. 2012;26(2):120–5. 22279045 10.1101/gad.182980.111 PMC3273836

[66] Yin T, Lallena MJ, Kreklau EL, Fales KR, Carballares S, Torrres R, et al. A novel CDK9 inhibitor shows potent antitumor efficacy in preclinical hematologic tumor models. Mol Cancer Ther. 2014;13(6):1442–56. 24688048 10.1158/1535-7163.MCT-13-0849

[67] Muntean AG, Hess JL. The pathogenesis of mixed-lineage leukemia. Annu Rev Pathol. 2012;7:283–301. 22017583 10.1146/annurev-pathol-011811-132434 PMC3381338

[68] Safaei S, Baradaran B, Hagh MF, Alivand MR, Talebi M, Gharibi T, et al. Double sword role of EZH2 in leukemia. Biomed Pharmacother. 2018;98:626–35. 29289837 10.1016/j.biopha.2017.12.059

[69] Argiropoulos B, Humphries RK. Hox genes in hematopoiesis and leukemogenesis. Oncogene. 2007;26(47):6766–76. 17934484 10.1038/sj.onc.1210760

[70] Lin C, Smith ER, Takahashi H, Lai KC, Martin-Brown S, Florens L, et al. AFF4, a component of the ELL/P-TEFb elongation complex and a shared subunit of MLL chimeras, can link transcription elongation to leukemia. Mol Cell. 2010;37(3):429–37. 20159561 10.1016/j.molcel.2010.01.026 PMC2872029

[71] Scholz A, Oellerich T, Hussain A, Lindner S, Luecking U, Walter AO, et al. BAY 1143572, a first-in-class, highly selective, potent and orally available inhibitor of PTEFb/CDK9 currently in Phase I, shows convincing anti-tumor activity in preclinical models of acute myeloid leukemia (AML). Cancer Res. 2016;76(14Supplement):3022.

[72] Lücking U, Scholz A, Lienau P, Siemeister G, Kosemund D, Bohlmann R, et al. Identification of Atuveciclib (BAY 1143572), the First Highly Selective, Clinical PTEFb/CDK9 Inhibitor for the Treatment of Cancer. ChemMedChem. 2017;12(21):1776–93. 28961375 10.1002/cmdc.201700447 PMC5698704

[73] Rahaman MH, Yu Y, Zhong L, Adams J, Lam F, Li P, et al. CDKI-73: an orally bioavailable and highly efficacious CDK9 inhibitor against acute myeloid leukemia. Invest New Drugs. 2019;37(4):625–35. 30194564 10.1007/s10637-018-0661-2

[74] Garcia-Cuellar MP, Füller E, Mäthner E, Breitinger C, Hetzner K, Zeitlmann L, et al. Efficacy of cyclin-dependent-kinase 9 inhibitors in a murine model of mixed-lineage leukemia. Leukemia. 2014;28(7):1427–35. 24445865 10.1038/leu.2014.40

[75] Baker A, Gregory GP, Verbrugge I, Kats L, Hilton JJ, Vidacs E, et al. The CDK9 Inhibitor Dinaciclib Exerts Potent Apoptotic and Antitumor Effects in Preclinical Models of MLL-Rearranged Acute Myeloid Leukemia. Cancer Res. 2016;76(5):1158–69. 26627013 10.1158/0008-5472.CAN-15-1070

[76] McCalmont H, Li KL, Jones L, Toubia J, Bray SC, Casolari DA, et al. Efficacy of combined CDK9/BET inhibition in preclinical models of MLL-rearranged acute leukemia. Blood Adv. 2020;4(2):296–300. 31971998 10.1182/bloodadvances.2019000586 PMC6988396

[77] Matsuoka M, Jeang K-T. Human T-cell leukaemia virus type 1 (HTLV-1) infectivity and cellular transformation. Nat Rev Cancer. 2007;7(4):270–80. 17384582 10.1038/nrc2111

[78] Narita T, Ishida T, Ito A, Masaki A, Kinoshita S, Suzuki S, et al. Cyclin-dependent kinase 9 is a novel specific molecular target in adult T-cell leukemia/lymphoma. Blood. 2017;130(9):1114–24. 28646117 10.1182/blood-2016-09-741983

[79] Johansson P, Dierichs L, Klein-Hitpass L, Bergmann AK, Möllmann M, Menninger S, et al. Anti-leukemic effect of CDK9 inhibition in T-cell prolymphocytic leukemia. Therapeutic Adv Hematol. 2020;11:2040620720933761. 10.1177/2040620720933761 PMC7570784 33117517

[80] O’Reilly A, Murphy J, Rawe S, Garvey M. Chronic Lymphocytic Leukemia: A Review of Front-line Treatment Options, With a Focus on Elderly CLL Patients. Clin Lymphoma Myeloma Leuk. 2018;18(4):249–56. 29477297 10.1016/j.clml.2018.02.003

[81] Pepper C, Lin TT, Pratt G, Hewamana S, Brennan P, Hiller L, et al. Mcl-1 expression has in vitro and in vivo significance in chronic lymphocytic leukemia and is associated with other poor prognostic markers. Blood. 2008;112(9):3807–17. 18599795 10.1182/blood-2008-05-157131

[82] Stevenson FK, Krysov S, Davies AJ, Steele AJ, Packham G. B-cell receptor signaling in chronic lymphocytic leukemia. Blood. 2011;118(16):4313–20. 21816833 10.1182/blood-2011-06-338855

[83] Kitada S, Zapata JM, Andreeff M, Reed JC. Protein kinase inhibitors flavopiridol and 7-hydroxy-staurosporine down-regulate antiapoptosis proteins in B-cell chronic lymphocytic leukemia. Blood. 2000;96(2):393–7. 10887097

[84] Mahoney E, Lucas DM, Gupta SV, Wagner AJ, Herman SE, Smith LL, et al. ER stress and autophagy: new discoveries in the mechanism of action and drug resistance of the cyclin-dependent kinase inhibitor flavopiridol. Blood. 2012;120(6):1262–73. 22740450 10.1182/blood-2011-12-400184 PMC3418721

[85] Desai AV, El-Bakkar H, Abdul-Hay M. Novel agents in the treatment of chronic lymphocytic leukemia: a review about the future. Clin Lymphoma Myeloma Leuk. 2015;15(6):314–22. 25445466 10.1016/j.clml.2014.09.007

[86] Stephens DM, Ruppert AS, Maddocks K, Andritsos L, Baiocchi R, Jones J, et al. Cyclophosphamide, alvocidib (flavopiridol), and rituximab, a novel feasible chemoimmunotherapy regimen for patients with high-risk chronic lymphocytic leukemia. Leuk Res. 2013;37(10):1195–9. 23867058 10.1016/j.leukres.2013.06.006 PMC3934299

[87] Nguyen L, Papenhausen P, Shao H. The Role of c-MYC in B-Cell Lymphomas: Diagnostic and Molecular Aspects. Genes (Basel). 2017;8(4):116. 10.3390/genes8040116 PMC5406863 28379189

[88] Coupland SE. The challenge of the microenvironment in B-cell lymphomas. Histopathology. 2011;58(1):69–80. 21261684 10.1111/j.1365-2559.2010.03706.x

[89] Chisholm KM, Bangs CD, Bacchi CE, Molina-Kirsch H, Cherry A, Natkunam Y. Expression profiles of MYC protein and MYC gene rearrangement in lymphomas. Am J Surg Pathol. 2015;39(3):294–303. 25581730 10.1097/PAS.0000000000000365

[90] Karube K, Campo E. MYC alterations in diffuse large B-cell lymphomas. Semin Hematol. 2015;52(2):97–106. 25805589 10.1053/j.seminhematol.2015.01.009

[91] Savage KJ, Johnson NA, Ben-Neriah S, Connors JM, Sehn LH, Farinha P, et al. MYC gene rearrangements are associated with a poor prognosis in diffuse large B-cell lymphoma patients treated with R-CHOP chemotherapy. Blood. 2009;114(17):3533–7. 19704118 10.1182/blood-2009-05-220095

[92] Eberhardy SR, Farnham PJ. Myc recruits P-TEFb to mediate the final step in the transcriptional activation of the cad promoter. J Biol Chem. 2002;277(42):40156–62. 12177005 10.1074/jbc.M207441200

[93] Kanazawa S, Soucek L, Evan G, Okamoto T, Peterlin BM. c-Myc recruits P-TEFb for transcription, cellular proliferation and apoptosis. Oncogene. 2003;22(36):5707–11. 12944920 10.1038/sj.onc.1206800

[94] Rowland T, Paiva C, Rowley J, Chen A, Drew L, Hurlin P, et al. Selective targeting cyclin-dependent kinase-9 (CDK9) downmodulates c-MYC and induces apoptosis in diffuse large B-cell lymphoma (DLBCL) cells. Blood. 2016;128(22):289.

[95] van de Donk N, Pawlyn C, Yong KL. Multiple myeloma. Lancet. 2021;397(10272):410–27. 33516340 10.1016/S0140-6736(21)00135-5

[96] Gojo I, Zhang B, Fenton RG. The cyclin-dependent kinase inhibitor flavopiridol induces apoptosis in multiple myeloma cells through transcriptional repression and down-regulation of Mcl-1. Clin Cancer Res. 2002;8(11):3527–38. 12429644

[97] McLaughlin RP, He J, van der Noord VE, Redel J, Foekens JA, Martens JWM, et al. A kinase inhibitor screen identifies a dual cdc7/CDK9 inhibitor to sensitise triple-negative breast cancer to EGFR-targeted therapy. Breast Cancer Res. 2019;21(1):77. 31262335 10.1186/s13058-019-1161-9 PMC6604188

[98] Lim SL, Xu L, Han BC, Shyamsunder P, Chng WJ, Koeffler HP. Multiple myeloma: Combination therapy of BET proteolysis targeting chimeric molecule with CDK9 inhibitor. PLoS ONE. 2020;15(6):e0232068. 32559187 10.1371/journal.pone.0232068 PMC7304913

[99] Borowczak J, Szczerbowski K, Ahmadi N, Szylberg Ł. CDK9 inhibitors in multiple myeloma: a review of progress and perspectives. Med Oncol. 2022;39(4):39. 35092513 10.1007/s12032-021-01636-1 PMC8800928

[100] Senderowicz AM. Flavopiridol: the first cyclin-dependent kinase inhibitor in human clinical trials. Invest New Drugs. 1999;17(3):313–20. 10665481 10.1023/a:1006353008903

[101] Khalil HS, Mitev V, Vlaykova T, Cavicchi L, Zhelev N. Discovery and development of Seliciclib. How systems biology approaches can lead to better drug performance. J Biotechnol. 2015;202:40–9. 25747275 10.1016/j.jbiotec.2015.02.032

[102] Karp JE, Garrett-Mayer E, Estey EH, Rudek MA, Smith BD, Greer JM, et al. Randomized phase II study of two schedules of flavopiridol given as timed sequential therapy with cytosine arabinoside and mitoxantrone for adults with newly diagnosed, poor-risk acute myelogenous leukemia. Haematologica. 2012;97(11):1736–42. 22733022 10.3324/haematol.2012.062539 PMC3487449

[103] Fekrazad HM, Verschraegen CF, Royce M, Smith HO, Chyi Lee F, Rabinowitz I. A phase I study of flavopiridol in combination with gemcitabine and irinotecan in patients with metastatic cancer. Am J Clin Oncol. 2010;33(4):393–7. 19884803 10.1097/COC.0b013e3181b2043f

[104] Stephenson JJ, Nemunaitis J, Joy AA, Martin JC, Jou YM, Zhang D, et al. Randomized phase 2 study of the cyclin-dependent kinase inhibitor dinaciclib (MK-7965) versus erlotinib in patients with non-small cell lung cancer. Lung Cancer. 2014;83(2):219–23. 24388167 10.1016/j.lungcan.2013.11.020

[105] Kumar SK, LaPlant B, Chng WJ, Zonder J, Callander N, Fonseca R, et al. Dinaciclib, a novel CDK inhibitor, demonstrates encouraging single-agent activity in patients with relapsed multiple myeloma. Blood. 2015;125(3):443–8. 25395429 10.1182/blood-2014-05-573741 PMC4296007

[106] Cassandri M, Fioravanti R, Pomella S, Valente S, Rotili D, Del Baldo G, et al. CDK9 as a Valuable Target in Cancer: From Natural Compounds Inhibitors to Current Treatment in Pediatric Soft Tissue Sarcomas. Front Pharmacol. 2020;11:1230. 32903585 10.3389/fphar.2020.01230 PMC7438590

[107] Zeidner JF, Karp JE. Clinical activity of alvocidib (flavopiridol) in acute myeloid leukemia. Leuk Res. 2015;39(12):1312–8. 26521988 10.1016/j.leukres.2015.10.010

[108] McClue SJ, Blake D, Clarke R, Cowan A, Cummings L, Fischer PM, et al. In vitro and in vivo antitumor properties of the cyclin dependent kinase inhibitor CYC202 (R-roscovitine). Int J Cancer. 2002;102(5):463–8. 12432547 10.1002/ijc.10738

[109] Meijer L, Borgne A, Mulner O, Chong JP, Blow JJ, Inagaki N, et al. Biochemical and cellular effects of roscovitine, a potent and selective inhibitor of the cyclin-dependent kinases cdc2, cdk2 and cdk5. Eur J Biochem. 1997;243(1–2):527–36. 9030781 10.1111/j.1432-1033.1997.t01-2-00527.x

[110] Mohapatra S, Chu B, Zhao X, Pledger WJ. Accumulation of p53 and reductions in XIAP abundance promote the apoptosis of prostate cancer cells. Cancer Res. 2005;65(17):7717–23. 16140939 10.1158/0008-5472.CAN-05-0347

[111] Whittaker SR, Mallinger A, Workman P, Clarke PA. Inhibitors of cyclin-dependent kinases as cancer therapeutics. Pharmacol Ther. 2017;173:83–105. 28174091 10.1016/j.pharmthera.2017.02.008 PMC6141011

[112] Parry D, Guzi T, Shanahan F, Davis N, Prabhavalkar D, Wiswell D, et al. Dinaciclib (SCH 727965), a novel and potent cyclin-dependent kinase inhibitor. Mol Cancer Ther. 2010;9(8):2344–53. 20663931 10.1158/1535-7163.MCT-10-0324

[113] Johnson AJ, Yeh YY, Smith LL, Wagner AJ, Hessler J, Gupta S, et al. The novel cyclin-dependent kinase inhibitor dinaciclib (SCH727965) promotes apoptosis and abrogates microenvironmental cytokine protection in chronic lymphocytic leukemia cells. Leukemia. 2012;26(12):2554–7. 22791353 10.1038/leu.2012.144 PMC3645353

[114] Sadowska M, Muvarak N, Lapidus RG, Sausville EA, Bannerji R, Gojo I. Single agent activity of the cyclin-dependent kinase (CDK) inhibitor dinaciclib (SCH 727965) in acute myeloid and lymphoid leukemia cells. Blood. 2010;116(21):3981.20634381

[115] Flynn J, Jones J, Johnson AJ, Andritsos L, Maddocks K, Jaglowski S, et al. Dinaciclib is a novel cyclin-dependent kinase inhibitor with significant clinical activity in relapsed and refractory chronic lymphocytic leukemia. Leukemia. 2015;29(7):1524–9. 25708835 10.1038/leu.2015.31 PMC4551390

[116] Hossain DMS, Javaid S, Cai M, Zhang C, Sawant A, Hinton M, et al. Dinaciclib induces immunogenic cell death and enhances anti-PD1-mediated tumor suppression. J Clin Invest. 2018;128(2):644–54. 29337311 10.1172/JCI94586 PMC5785250

[117] Conroy A, Stockett DE, Walker D, Arkin MR, Hoch U, Fox JA, et al. SNS-032 is a potent and selective CDK 2, 7 and 9 inhibitor that drives target modulation in patient samples. Cancer Chemother Pharmacol. 2009;64(4):723–32. 19169685 10.1007/s00280-008-0921-5

[118] Tong WG, Chen R, Plunkett W, Siegel D, Sinha R, Harvey RD, et al. Phase I and pharmacologic study of SNS-032, a potent and selective Cdk2, 7, and 9 inhibitor, in patients with advanced chronic lymphocytic leukemia and multiple myeloma. J Clin Oncol. 2010;28(18):3015–22. 20479412 10.1200/JCO.2009.26.1347 PMC4979218

[119] Olson CM, Jiang B, Erb MA, Liang Y, Doctor ZM, Zhang Z, et al. Pharmacological perturbation of CDK9 using selective CDK9 inhibition or degradation. Nat Chem Biol. 2018;14(2):163–70. 29251720 10.1038/nchembio.2538 PMC5912898

[120] Guhan SM, Shaughnessy M, Rajadurai A, Taylor M, Kumar R, Ji Z, et al. The Molecular Context of Vulnerability for CDK9 Suppression in Triple Wild-Type Melanoma. J Invest Dermatol. 2021;141(8):2018–e274. 33745909 10.1016/j.jid.2020.12.035 PMC8316294

[121] Luecking U, Scholz A, Kosemund D, Bohlmann R, Briem H, Lienau P, et al. Abstract 984: Identification of potent and highly selective PTEFb inhibitor BAY 1251152 for the treatment of cancer: from p.o. to i.v. application via scaffold hops. Cancer Res. 2017;77:984.

[122] Johansson P, Dierichs L, Klein-Hitpass L, Bergmann AK, Möllmann M, Menninger S, et al. Anti-leukemic effect of CDK9 inhibition in T-cell prolymphocytic leukemia. Ther Adv Hematol. 2020;11:2040620720933761. 33117517 10.1177/2040620720933761 PMC7570784

[123] Ruff JP, Kretz AL, Kornmann M, Henne-Bruns D, Lemke J, Traub B. The Novel, Orally Bioavailable CDK9 Inhibitor Atuveciclib Sensitises Pancreatic Cancer Cells to TRAIL-induced Cell Death. Anticancer Res. 2021;41(12):5973–85. 34848451 10.21873/anticanres.15416

[124] Cidado J, Boiko S, Proia T, Ferguson D, Criscione SW, San Martin M, et al. AZD4573 Is a Highly Selective CDK9 Inhibitor That Suppresses MCL-1 and Induces Apoptosis in Hematologic Cancer Cells. Clin Cancer Res. 2020;26(4):922–34. 31699827 10.1158/1078-0432.CCR-19-1853

[125] Boiko S, Proia T, San Martin M, Gregory GP, Wu MM, Aryal N, et al. Targeting Bfl-1 via acute CDK9 inhibition overcomes intrinsic BH3-mimetic resistance in lymphomas. Blood. 2021;137(21):2947–57. 33259592 10.1182/blood.2020008528 PMC8160501

[126] Alcon C, Manzano-Muñoz A, Montero J. A New CDK9 Inhibitor on the Block to Treat Hematologic Malignancies. Clin Cancer Res. 2020;26(4):761–3. 31843752 10.1158/1078-0432.CCR-19-3670

[127] Rule S, Kater AP, Brümmendorf TH, Fegan C, Kaiser M, Radford JA, et al. A phase 1, open-label, multicenter, non-randomized study to assess the safety, tolerability, pharmacokinetics, and preliminary antitumor activity of AZD4573, a potent and selective CDK9 inhibitor, in subjects with relapsed or refractory hematological malignancies. J Clin Oncol. 2018;36(15suppl):TPS7588–TPS.

[128] Zhang H, Huang C, Gordon J, Yu S, Morton G, Childers W, et al. MC180295 is a highly potent and selective CDK9 inhibitor with preclinical in vitro and in vivo efficacy in cancer. Clin Epigenetics. 2024;16(1):3. 38172923 10.1186/s13148-023-01617-3 PMC10765884

[129] Egloff S. CDK9 keeps RNA polymerase II on track. Cell Mol Life Sci. 2021;78(14):5543–67. 34146121 10.1007/s00018-021-03878-8 PMC8257543

[130] van der Noord VE, McLaughlin RP, Karuntu JS, He J, Timmermans AM, Basnet SKC, et al. Disrupting CDK9 activity suppresses triple-negative breast cancer and is enhanced by EGFR Inhibition. Cell Oncol (Dordr). 2026;49(1):20. 41505030 10.1007/s13402-025-01154-6 PMC12783313

[131] Xiao L, Liu Y, Chen H, Shen L. Targeting CDK9 with selective inhibitors or degraders in tumor therapy: an overview of recent developments. Cancer Biol Ther. 2023;24(1):2219470. 37272701 10.1080/15384047.2023.2219470 PMC10243401

[132] Hu C, Shen L, Zou F, Wu Y, Wang B, Wang A, et al. Predicting and overcoming resistance to CDK9 inhibitors for cancer therapy. Acta Pharm Sin B. 2023;13(9):3694–707. 37719386 10.1016/j.apsb.2023.05.026 PMC10502288

[133] Lu H, Xue Y, Yu GK, Arias C, Lin J, Fong S, et al. Compensatory induction of MYC expression by sustained CDK9 inhibition via a BRD4-dependent mechanism. Elife. 2015;4:e06535. 26083714 10.7554/eLife.06535 PMC4490784

[134] Liu R. Brd4-dependent CDK9 expression induction upon sustained pharmacological inhibition of P-TEFb kinase activity. Biochem Biophys Res Commun. 2023;671:75–9. 37295357 10.1016/j.bbrc.2023.05.114

[135] Padmanabhan J, Saha B, Powell C, Mo Q, Perez BA, Chellappan S. Inhibitors Targeting CDK9 Show High Efficacy against Osimertinib and AMG510 Resistant Lung Adenocarcinoma Cells. Cancers. 2021;13(15):3906. 34359807 10.3390/cancers13153906 PMC8345430

[136] Thieme E, Bruss N, Sun D, Dominguez EC, Coleman D, Liu T, et al. CDK9 inhibition induces epigenetic reprogramming revealing strategies to circumvent resistance in lymphoma. Mol Cancer. 2023;22(1):64. 36998071 10.1186/s12943-023-01762-6 PMC10061728

[137] Yan F, Jiang V, Jordan A, Che Y, Liu Y, Cai Q, et al. The HSP90-MYC-CDK9 network drives therapeutic resistance in mantle cell lymphoma. Experimental Hematol Oncol. 2024;13(1):14. 10.1186/s40164-024-00484-9 PMC10848414 38326887

[138] Satta T, Grant S. Enhancing venetoclax activity in hematological malignancies. Expert Opin Investig Drugs. 2020;29(7):697–708. 32600066 10.1080/13543784.2020.1789588 PMC7529910

[139] George B, Chowdhury SM, Hart A, Sircar A, Singh SK, Nath UK et al. Ibrutinib Resistance Mechanisms and Treatment Strategies for B-Cell lymphomas. Cancers (Basel). 2020;12(5):1328. 10.3390/cancers12051328 PMC7281539 32455989

[140] Alvarado-Valero Y, Cook RJ, Dinner SN, Keng M, Begna KH, Javidi-Sharifi N, et al. The oral CDK9 inhibitor voruciclib combined with venetoclax for patients with relapsed/refractory acute myeloid leukemia. Blood Neoplasia. 2025;2(3):100108. 40809193 10.1016/j.bneo.2025.100108 PMC12343358

[141] Tran S, Sipila P, Frigault MM, Stelte-Ludwig B, Johnson AJ, Birkett J, et al. Enitociclib, a selective CDK9 inhibitor: in vitro and in vivo preclinical studies in multiple myeloma. Blood Neoplasia. 2025;2(1):100050. 40546728 10.1016/j.bneo.2024.100050 PMC12182852

[142] Fultang N, Schwab AM, McAneny-Droz S, Heiser D, Scherle PA, Bhagwat N. PRT2527, a Novel Highly Selective Cyclin-Dependent Kinase 9 (CDK9) Inhibitor, Has Potent Antitumor Activity in Combination with BTK and BCL2 Inhibition in Various Lymphoid Malignancies. Blood. 2023;142(Supplement 1):5783.

[143] Scheffold A, Jebaraj BMC, Stilgenbauer S, Venetoclax. Targeting BCL2 in Hematological Cancers. Recent Results Cancer Res. 2018;212:215–42. 30069633 10.1007/978-3-319-91439-8_11

[144] Bhutada I, Khambati F, Cheng SY, Tiek DM, Duckett D, Lawrence H, et al. CDK7 and CDK9 inhibition interferes with transcription, translation, and stemness, and induces cytotoxicity in GBM irrespective of temozolomide sensitivity. Neuro Oncol. 2024;26(1):70–84. 37551745 10.1093/neuonc/noad143 PMC10768977

[145] Pimentel JM, Zhou JY, Wu GS. The Role of TRAIL in Apoptosis and Immunosurveillance in Cancer. Cancers (Basel). 2023;15(10):2752. 10.3390/cancers15102752 PMC10216286 37345089

[146] Ohnstad HO, Paulsen EB, Noordhuis P, Berg M, Lothe RA, Vassilev LT, et al. MDM2 antagonist Nutlin-3a potentiates antitumour activity of cytotoxic drugs in sarcoma cell lines. BMC Cancer. 2011;11:211:1–11. 21624110 10.1186/1471-2407-11-211 PMC3128006

[147] Ma L, Xie L, Wang Y, Guan X, Zhang Y, Guo H, et al. Discovery of dCDK9-202 as a Highly Potent and Selective PROTAC CDK9 Degrader with Strong In Vivo Antitumor Activity. J Med Chem. 2025;68(20):21172–86. 41066447 10.1021/acs.jmedchem.5c01111 PMC12557382

